# Ets-1 transcription factor regulates glial cell regeneration and function in planarians

**DOI:** 10.1242/dev.201666

**Published:** 2023-09-14

**Authors:** Bidushi Chandra, Matthew G. Voas, Erin L. Davies, Rachel H. Roberts-Galbraith

**Affiliations:** ^1^ Department of Cellular Biology, University of Georgia, Athens, GA 30602, USA; ^2^ Center for Cancer Research, National Cancer Institute, National Institutes of Health, Frederick, MD 21702, USA

**Keywords:** Planarian, Glia, *S. mediterranea*, Regeneration, Nervous system, ETS

## Abstract

Glia play multifaceted roles in nervous systems in response to injury. Depending on the species, extent of injury and glial cell type in question, glia can help or hinder the regeneration of neurons. Studying glia in the context of successful regeneration could reveal features of pro-regenerative glia that could be exploited for new human therapies. Planarian flatworms completely regenerate their nervous systems after injury – including glia – and thus provide a strong model system for exploring glia in the context of regeneration. Here, we report that planarian glia regenerate after neurons, and that neurons are required for correct glial numbers and localization during regeneration. We also identify the planarian transcription factor-encoding gene *ets-1* as a key regulator of glial cell maintenance and regeneration. Using *ets-1* (RNAi) to perturb glia, we show that glial loss is associated with altered neuronal gene expression, impeded animal movement and impaired nervous system architecture – particularly within the neuropil. Importantly, our work reveals the inter-relationships of glia and neurons in the context of robust neural regeneration.

## Teaser

**Summary:** Perturbation of glia with *ets-1*(RNAi) alters neuronal gene expression, impedes animal movement and impairs nervous system architecture in planarians.

## INTRODUCTION

Glial cells are a heterogenous group of non-neuronal cells within many animal nervous systems. Glial cells within the mammalian central nervous system (CNS) include astrocytes, oligodendrocytes and microglia. Meanwhile, major glial types in the mammalian peripheral nervous system (PNS) include Schwann cells and satellite cells (Allen and Lyons, 2018; Jessen, 2004). Glial cells have been reported in most, but not all, bilaterian taxa studied (Hartline, 2011; Ortega and Olivares-Bañuelos, 2020). Molecularly, glial cells often express *glial fibrillary acidic protein* (GFAP), *glutamine synthetase* (GS) and/or *excitatory amino acid transporter* (EAAT), but these markers are not universal across species (Hartline, 2011). Functionally, glial cells play important and diverse roles in the development, maintenance and activity of the nervous system across bilaterians. Glial roles include: regulating neural cell numbers and migration; promoting axon guidance; maintaining ionic homeostasis; mediating neurotransmitter uptake; facilitating synapse architecture; and remodeling neural circuitry (Allen and Lyons, 2018; Jäkel and Dimou, 2017; Jessen, 2004; Oikonomou and Shaham, 2011; Shaham, 2015; Yildirim et al., 2019).

In regeneration, glial cells play dynamic roles that depend on the species, location, extent and duration of the injury. For example, mammalian astrocytes and microglia respond to injury through ‘reactive gliosis’, which can lead to formation of a ‘glial scar’ (Adams and Gallo, 2018; Anderson et al., 2016; Burda and Sofroniew, 2014; Escartin et al., 2021; Gallo and Deneen, 2014; Pekny et al., 2014). Glial scarring can promote neuronal survival, but can also limit axonal regeneration (Anderson et al., 2016; Myer, 2006; Rolls et al., 2009; Silver and Miller, 2004). In contrast, glial scarring does not occur in fish and insects owing to the presence of bridging glia in the zebrafish spinal cord (Goldshmit et al., 2012; Mokalled et al., 2016) and phagocytic ensheathing glia in the adult *Drosophila* neuropil (Doherty et al., 2009; Purice et al., 2017). Glial roles in the context of successful regeneration are only beginning to be uncovered, partly due to the limited regenerative capacity of many traditional model organisms (Alesci et al., 2022; Silver et al., 2015; Tanaka and Ferretti, 2009).

Freshwater flatworms called planarians undergo whole-body regeneration without scarring, including *de novo* regrowth and rewiring of the entire brain. Genes that include *intermediate filament* (IF-1), *calamari* and *estrella* mark glia present in the planarian nervous system, opening for the first time an opportunity to identify glia and to explore glial biology in an organism with complete regenerative capacity (Roberts-Galbraith et al., 2016; Wang et al., 2016). Planarian glia express genes that encode proteins for neurotransmitter uptake and metabolism (e.g. *solute carrier 1a-5*/*EAAT* and *glutamine synthetase-1*), indicating an overlap in function with astrocytes in the mammalian CNS (Roberts-Galbraith et al., 2016; Wang et al., 2016). Previous work also determined that the transcription factor *forkhead box protein factor-1* (Scimone et al., 2018) and Hedgehog signaling from ventral-medial neurons impact glial gene expression in planarians (Currie et al., 2016; Wang et al., 2016), although the consequences of changes in glial gene expression are not known. Furthermore, single-cell sequencing atlases indicate that planarian glia share some gene expression features with additional cell types that express *cathepsin*, including pigment cells, parenchymal cells and other uncharacterized cell types (Fincher et al., 2018; Plass et al., 2018). Transcriptional similarity between *cathepsin^+^* cell types has been interpreted as a lineage-based relationship, although this has not yet been experimentally shown. Many fundamental aspects of planarian glial biology remain unexplored, including how glia regenerate and what roles, if any, they play in the planarian nervous system during homeostasis and regeneration.

ETS transcription factors regulate gliogenesis in vertebrates and invertebrates (Kiyota et al., 2007; Klämbt, 1993; Kola et al., 1993). *Drosophila pointed*, which encodes an ETS transcription factor, is necessary and sufficient for longitudinal and midline glial differentiation (Klaes et al., 1994). In *Xenopus*, *ets-1* directly regulates radial glial formation and promotes neuron-glia interaction during embryogenesis (Kiyota et al., 2007; Klämbt, 1993). Human and mouse *ETS1*/*Ets1* are expressed in cortical astrocytes, and are involved in astrocyte differentiation (Amouyel et al., 1988; Fleischman et al., 1995). In planarians, previous studies indicate that *ets-1* plays roles in several cell types, including pigment cells, glial cells and other uncharacterized *cathepsin^+^* cells (Dubey et al., 2022; He et al., 2017); however, a definitive function for *ets-1* in glia has not been fully established.

In this study, we determined that the transcription factor Ets-1 promotes maintenance of existing glial cells in uninjured tissues and regeneration of new glial cells in the planarian *Schmidtea mediterranea*. Furthermore, using *ets-1*(RNAi) to perturb glia, we investigated potential roles for planarian glia for the first time. We determined that *ets-1*(RNAi) non-cell-autonomously impacts neuronal gene expression, neuropil size and animal movement. Taken together, our work demonstrates that planarian *ets-1* plays a conserved and crucial role in glial cells during regeneration and tissue homeostasis in planarians. In parallel, this work explores spatiotemporal and functional relationships between planarian glial cells and neurons during regeneration and embryogenesis, revealing that planarian neurons also promote glial regeneration.

## RESULTS

### Planarian glial cells arise after neurons

**Fig. 1.**
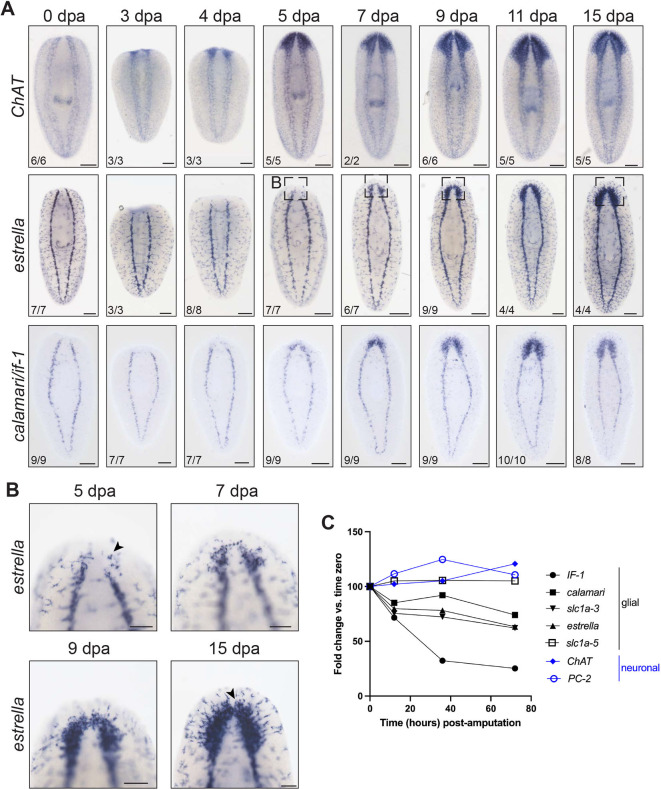
**Planarian glial cells regenerate after neurons.** (A) *In situ* hybridization regeneration timeline of neurons (top), marked by *choline acetyltransferase* (*ChAT*), and glial cells (bottom), marked by *estrella* or pooled *if-1*/*calamari*. (B) Higher magnification of *estrella* expression in head blastema at 5, 7, 9 and 15 dpa. Arrowheads indicate round *estrella*^+^ cells (5 dpa) that progress to stellate morphology (15 dpa). (C) RNA-seq of tail fragments regenerating head tissue illustrates glial and neuronal marker transcript levels at early time points post-amputation (re-analysis of data from Roberts-Galbraith et al., 2016). Planarian glial markers are downregulated in the first 72 h post-amputation. Ventral views, anterior upwards. Scale bars: 200 µm in A; 100 µm in B.

In vertebrate and *Drosophila* development, neurogenesis precedes gliogenesis (Anthony et al., 2004; Barnabé-Heider et al., 2005; Bayraktar and Doe, 2013; Klämbt and Goodman, 1991; Viktorin et al., 2011). We reasoned that understanding the sequence of neuronal and glial development and regeneration in planarians could help form testable hypotheses about glial cell specification and function. We first amputated planarians pre-pharyngeally and fixed animals at several time points to establish a timeline of neuronal and glial regeneration. Using a marker of cholinergic neurons, *choline acetyltransferase* (*ChAT*) (Nishimura et al., 2010), we observed re-establishment of neurons within a primordial brain around 3 days post-amputation (dpa) and clear brain organization at 5 dpa (Fig. 1A, top). Our results were consistent with previous reports that new neurons are born from 2 dpa onwards (Fig. S1A,B) (Cebrià et al., 2002b; Inoue et al., 2004). The expression pattern of *ChAT* in the brain remained comparable from 5 dpa onwards.

In contrast to *ChAT*, the earliest appearance of *estrella^+^* glial cells in the newly regenerated head occurred between 4 and 5 dpa (Fig. 1A, middle) (Roberts-Galbraith et al., 2016). Round *estrella*^+^ cells initially re-appeared in small numbers; cell number increased over time and cells progressively adopted stellate morphology (Fig. 1B). By 15 dpa, the distribution of *estrella*^+^ cells appeared identical to that in uninjured planarians. We confirmed this timeline for glial cell regeneration using two additional glial markers: *intermediate filament-1* (*if-1*) (Roberts-Galbraith et al., 2016; Wang et al., 2016) and *calamari* (Wang et al., 2016) (Fig. 1A, bottom; Fig. S1A). *if-1* is also downregulated after amputation according to RNA-sequencing (RNA-seq) data and we confirmed that other glial markers were transiently downregulated at early time points after injury (Fig. 1C) (Roberts-Galbraith et al., 2016). Taken together, our results show that the planarian glial cells respond to injury by changing gene expression and regenerate in new tissue after neurons.

**Fig. 2.**
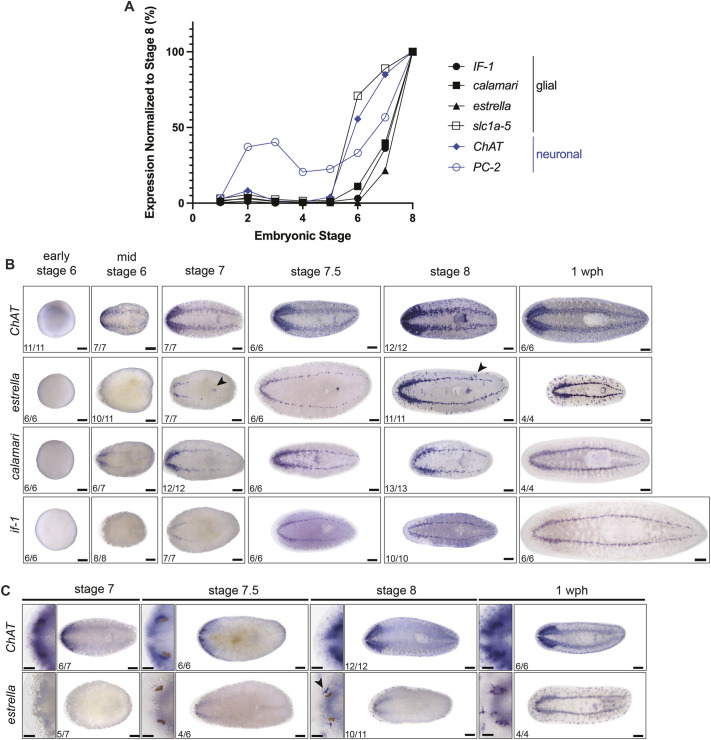
**Planarian glial cells develop after neurons during embryogenesis.** (A) Single embryo RNA-sequencing shows expression of neuronal and glial markers (re-analysis of data from Davies et al., 2017). (B) *In situ* hybridization of neuronal (*ChAT*) and glial (*if-1*, *calamari* and *estrella*) markers in planarian embryos (early S6, mid-S6, S7, S7.5 and S8) and juveniles (1 week post-hatching); ventral views. Arrowheads indicate *estrella* expression in mouth (S7) and peripheral nervous system (S8). (C) *In situ* hybridization of *ChAT* and *estrella* on staged embryos; dorsal views. Higher magnification images show expression near and within the eyespot. *estrella* expression near the eye appears at S8 (arrowhead); *ChAT* expression, in contrast, is seen as early as S7. Anterior is leftwards. Scale bars: 200 µm; 50 µm (higher magnification images).

Next, we asked whether the temporal order of cell birth holds true in embryogenesis. Development of the adult nervous system begins during stage 5 (S5), with the expression of transcription factor-encoding genes with roles in neuronal subtype specification (Davies et al., 2017). Genes required for differentiated neuron function, including terminal selector genes required for neuronal subtype maintenance and genes involved in synapsis and neurotransmission, show enriched expression during stage 6 (S6), stage 7 (S7) and stage 8 (S8) (Davies et al., 2017). Expression of the neuronal marker *pc-2* was detected by single embryo bulk RNA-seq as early as stage 2 (S2) (Fig. 2A). We did not detect robust expression of glial markers *if-1*, *calamari* and *estrella* by bulk RNA-Seq before S7, apart from *EAAT*/*slc1a-5* (Fig. 2A). However, single cell transcriptomic data from adult asexual planarians suggest that *EAAT* is also expressed in muscle and other *cathepsin*^+^ cells (Fincher et al., 2018; Plass et al., 2018).

*In situ* hybridization was performed to examine spatiotemporal expression patterns for neuronal- and glial-enriched transcripts on staged planarian embryos and juveniles (1 week post-hatching; wph). By *in situ* hybridization, *ChAT*^+^ neurons can be seen at S6 (Fig. 2B) (Davies et al., 2017). Neuronal markers *synaptotagmin* (*syt1-1*) and *pc-2* also appeared during S6 (Fig. S1C). By the end of S7, neuronal markers highlighted well-developed brain and ventral nerve cord (VNC) structures that were nearly contiguous and extended to the posterior end of the embryo (Fig. 2B, Fig. S1C). Peripheral neurons were evident in both *ChAT* and *syt1-1 in situ* hybridization during stage S6, and photoreceptor neurons were evident by S7 (Davies et al., 2017) (Fig. 2C, Fig. S1C,D). In planarians, as in other organisms, neurogenesis initiated in the anterior with the formation of the brain primordia, followed by VNC formation, which again showed early anterior bias (Fig. 2B, Fig. S1C).

We next examined expression of glial markers during embryonic development. Expression of markers specific for differentiated glial cells began at S6-S7 (Davies et al., 2017) (Fig. 2A). Expression of *calamari* in the CNS initiated first and was detected by whole-mount *in situ* hybridization in the brain primordia and anterior domain of the developing VNC at mid-S6 (Fig. 2B). *if-1* and *estrella* showed expression in the brain primordia and developing VNC in the anterior half of the embryo during S7 and in the posterior by S7.5 (Fig. 2B). *estrella* was expressed near the mouth beginning at S7, and around the eyes and in putative PNS glial cells in S8 hatchlings (Fig. 2B,C). All PNS glia and those around sensory structures such as the eye appeared later than neurons. Expression of peripheral glial marker genes was heavily biased towards the ventral side of the embryos at all stages assayed (Fig. 2B,C, Fig. S1C). Taken together, we concluded that gliogenesis occurs after neurogenesis during embryogenesis and adult regeneration, which concurs with the relative order of cell birth in other animal species.

### Glial regeneration in the nervous system depends on neurons

**Fig. 3.**
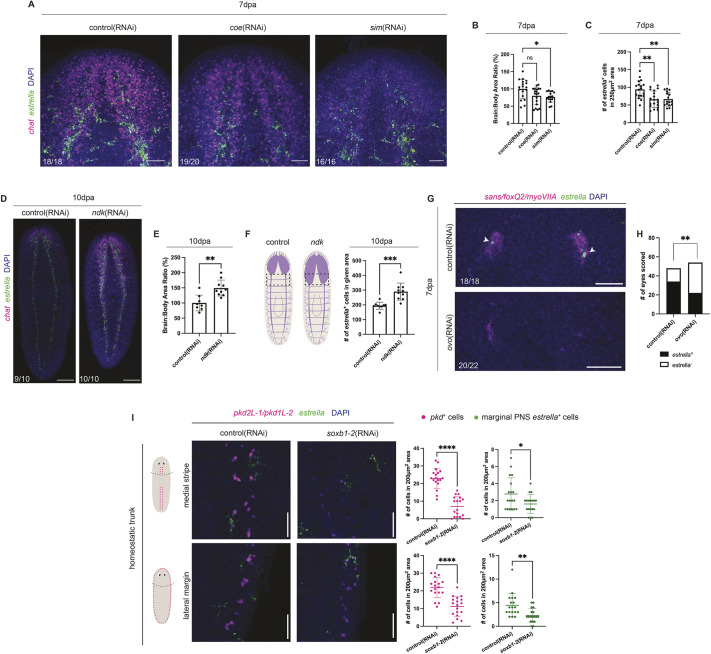
**The presence of *estrella*^+^ cells is neuron dependent.** (A) Fluorescence *in situ* hybridization of regenerated control, *coe*(RNAi) and *sim*(RNAi) animals, detecting *ChAT* (neurons, magenta) and *estrella* (glia, green) transcripts and stained using DAPI (nuclei, blue). (B) Quantification of brain-to-body ratio [normalized to control (100%)]. Unpaired *t*-test with Welch's correction. (C) Quantification of *estrella*^+^ cells in the head region in 250 µm^2^ areas. Unpaired *t*-test with Welch's correction. (D) Fluorescence *in situ* hybridization of regenerated control and *ndk*(RNAi) animals detecting *ChAT* (magenta) and *estrella* (green), and stained with DAPI (blue). (E) Quantification of normalized brain-to-body ratio. Unpaired *t*-test with Welch's correction. (F) Quantification of *estrella*^+^ cells in expanded posterior of the brain (depicted in illustration) in control and *ndk*(RNAi) animals. Unpaired *t*-test with Welch's correction. (G) Fluorescence *in situ* hybridization of regenerated control and *ovo*(RNAi) animals detecting pooled *sans/foxQ2/myoVIIA* (photoreceptor neurons, magenta) and *estrella* (green), and stained with DAPI (blue). Arrowheads indicate *estrella*^+^ cells in or near the eyespot. (H) Quantification of *estrella^+^* (black) or *estrella^−^* (white) eyespots after control or *ovo*(RNAi). Fisher's exact test. (I) Fluorescence *in situ* hybridization of control and *soxB1-2*(RNAi) animals detecting pooled *pkd2L-1*/*pkd1L-2* (sensory neurons, magenta) and *estrella* (green), and stained with DAPI (blue) in medial stripe and lateral margins in non-regenerated trunk tissue (dorsal view). Quantification of *pkd*^+^ or *estrella*^+^ cells in respective regions illustrated on the left. Unpaired *t*-test with Welch's correction (*n*=18). *P≤0.05, **P≤0.01, ***P≤0.001, ****P≤0.0001; ns, not significant. Data are mean±s.d. Scale bars: 50 µm in A,G; 200 µm in D,I.

In many organisms, the interactions between neurons and glia during development are dynamic and reciprocal. Neurons can act as a ‘blueprint’ for glial cell development by regulating the migration, survival and proliferation of glial cells (Allen and Lyons, 2018). The birth order we established for planarian glia and neurons led us to seek ways to test whether glia depend on neurons for birth or final location. *estrella*^+^ glial cells are present throughout the CNS, near the photoreceptor neurons of the eyespots, and among the ciliated sensory neurons along the dorsal midline and lateral margins of the body (Fig. 3, Fig. S2).

To investigate whether *estrella*^+^ cell regeneration in the CNS depends on neurons, we knocked down regulators of neuronal regeneration, *coe* and *sim* (Cowles et al., 2013, 2014), and performed fluorescence *in situ* hybridization to detect neuronal marker *ChAT* and glial marker *estrella*. As previously reported, regenerating *coe*(RNAi) and *sim*(RNAi) animals have reduced expression of *ChAT* (Fig. 3A,B). We saw significantly reduced *estrella*^+^ cells in the brains of both *coe*(RNAi) and *sim*(RNAi) animals (Fig. 3C). Next, we asked whether the expansion of the brain region would result in increased *estrella*^+^ cells. Previous work has shown that knockdown of *nou-darake* (*ndk*) causes posterior expansion of the brain (Fig. 3D,E) (Cebrià et al., 2002a). We performed *ndk*(RNAi) to assess *estrella*^+^ cells in the expanded area. Compared with control, we observed a significant increase in *estrella*^+^ cells in the expanded brain tissue (Fig. 3F). Our data suggest that CNS neurons could promote glial regeneration and/or localization to the brain.

To investigate whether *estrella*^+^ cells depend on photoreceptors or pigment cells in the eye, we knocked down *ovo* to specifically reduce eyespot regeneration (Lapan and Reddien, 2012). As previously reported, *ovo*(RNAi) caused reduced expression of pooled photoreceptor neuron markers *sans*/*foxQ2*/*myoVIIA* (Fig. 3G; Fig. S2A,B). We examined *estrella* expression in cells adjacent to the eyespots (Fig. 3G,H) (Roberts-Galbraith et al., 2016). We saw significant reduction of dorsal *estrella*^+^ cells present in the head after *ovo*(RNAi) (Fig. 3H). Our data suggest that the presence of photoreceptor neurons or pigment cup cells fosters localization of *estrella*^+^ glial cells nearby.

We finally sought to determine whether regeneration and maintenance of PNS glia depend on sensory neurons present along the medial and lateral dorsal surfaces. Transcription factor *soxb1-2* is specifically required for regeneration of many sensory neurons that function in these regions (Ross et al., 2018). We thus performed head amputations on *soxB1-2*(RNAi) animals, and examined *estrella* expression. As previously shown, *pkd2L-1/1L-2*^+^ neurons decreased in number after *soxB1-2*(RNAi) in the medial stripes and the lateral margin of the body (Fig. 3I; Fig. S2C,D) (Ross et al., 2018). In both regenerated and non-regenerating tissue, we observed a decrease in the number of *estrella*^+^ cells in medial and lateral regions; however, the differences were only statistically significant in the homeostatic tissue (Fig. 3I, Fig. S2C,D). We do acknowledge that perduring *pkd*^+^ and *soxb1-2*-independent neurons present in these locations may still permit glial localization. Additionally, *soxb1-2*(RNAi) also results in reduced brain size, and we observed reduced numbers of CNS *estrella*^+^ cells (Fig. S2E-G), reinforcing our previous observations that neurons promote regeneration of glial cells in the brain.

Taken together, our data show that perturbation of neurons within the CNS and PNS leads to reduction in local *estrella^+^* cells. Our results strongly suggest that population of the nervous system with glia during regeneration may be promoted by antecedent neuronal cell types.

### Putative transcription factor *ets-1* affects glial gene expression

Beyond the impact of neurons on glial regeneration, we also wished to identify genes required cell-autonomously for glial regeneration. Work in several organisms established roles for ETS-family transcription factors in glial cell identity (Amouyel et al., 1988; Fleischman et al., 1995; Kiyota et al., 2007; Klaes et al., 1994; Klämbt, 1993). We used phylogenetic analysis to show that *S. mediterranea* Ets-1 is similar to *D. melanogaster* and *C. salei* Pointed (Chen et al., 1992; Klämbt, 1993; Pribyl et al., 1988), and to mammalian and *Xenopus* Ets-1 (Fig. S3) (Slupsky et al., 1998; Stiegler et al., 1990; Watson et al., 1988).

**Fig. 4.**
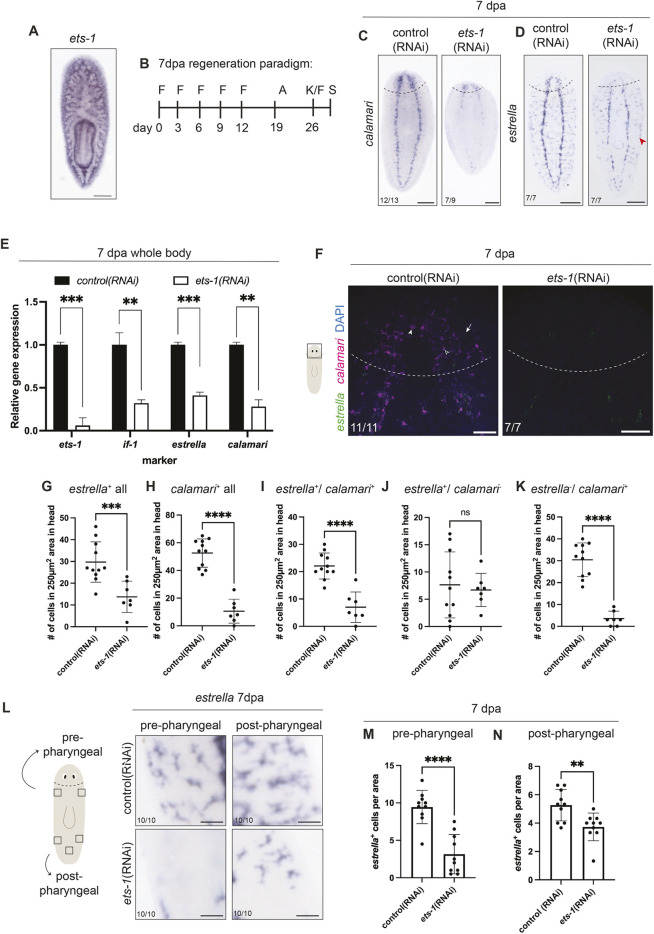
***ets-1* affects glial gene expression.** (A) Whole-mount *in situ* hybridization of *ets-1* in uninjured untreated animals. (B) RNAi feeding paradigm for *ets-1*(RNAi). Feeding (F), amputation (A), kill/fix (K/F) and staining (S) are indicated. (C,D) *In situ* hybridization of *ets-1*(RNAi) regenerated animals detecting *calamari* or *estrella* expression. Red arrowhead indicates reduced peripheral *estrella*^+^ cells. (E) RT-qPCR used to detect levels of *ets-1*, *if-1*, *estrella* and *calamari* transcripts after RNAi and regeneration. Unpaired *t*-test. Data are mean±s.e.m. (F) Fluorescence *in situ* hybridization for *calamari* (magenta) and *estrella* (green), and stained with DAPI (cell nuclei, blue) in newly regenerated heads in control and *ets-1*(RNAi) animals. White arrowhead indicates *cali*^+^/*estrella*^−^; black arrowhead indicated *cali*^+^/*estrella*^+^; arrow indicates *cali*^−^/*estrella*^+^. (G-K) Quantification of glia markers in 250 µm^2^ areas within head blastemas from F. Unpaired *t*-test with Welch's correction. Data are mean±s.d. (L-N) Illustration showing 200 µm^2^ squares used to quantify PNS *estrella*^+^ cells. Images and quantifications show reduced peripheral *estrella^+^* cells in *ets-1*(RNAi) animals (see also red arrowhead in D). Unpaired *t*-test with Welch's correction. Dashed lines indicate amputation sites. Data are mean±s.d. **P≤0.01, ***P≤0.001, ****P≤0.0001; ns, not significant. Scale bar: 200 µm in A,C,D,F; 50 µm in L.

Planarian *ets-1* is expressed widely throughout the mesenchyme (Fig. 4A), and has been reported to play roles in specification and maintenance of pigment cells and other *cathepsin^+^* cell types (Dubey et al., 2022; He et al., 2017). Previous studies on planarian *ets-1* reported either no impact on *estrella* expression (He et al., 2017) or decreased *calamari* expression in the head (Dubey et al., 2022). To uncover the full impact of *ets-1* on glial cells, we optimized an RNAi paradigm by adjusting numbers and dose of dsRNA feedings (Fig. 4B, Fig. S4A). After optimization, we performed *ets-1*(RNAi) and examined glia with multiple markers (Fig. 4C,D). *ets-1*(RNAi) worms exhibited reduced *calamari* expression throughout the body compared with controls, in regenerated and uninjured tissue (Fig. 4C, Fig. S4F,G) (Wang et al., 2016). Similarly, *ets-1*(RNAi) animals showed a reduction of *estrella* expression at 7 dpa and during homeostasis (Fig. 4D, Fig. S4H). We observed three distinct differences in *estrella* expression in *ets-1*(RNAi) animals. In both regenerating and uninjured animals, we observed decreased *estrella^+^* cell number in the newly regenerated head (Fig. 4D, Fig. S4B,C); gaps in *estrella^+^* signal in the VNC (Fig. 4D; Fig. S4D,E,I,J); and reduction of peripheral *estrella^+^* cell number (Fig. 4D, red arrowhead, L-N, Fig. S4K,L). Interestingly, the reduction of *calamari* expression was strongest in the VNC and more dramatic than the reduction of *estrella* (Fig. 4C, Fig. S4G). We further quantified relative expression of *if-1*, *estrella* and *calamari* transcripts via RT-qPCR and observed significant reduction in all glial transcripts (59-72% reduction; Fig. 4E) in *ets-1*(RNAi) animals. Taken together, our data indicate that *ets-1* promotes *calamari*^+^ and *estrella*^+^ cell maintenance in pre-existing tissue, as well as during regeneration.

Our previous data showed that changes in *estrella* and *calamari* expression after *ets-1*(RNAi) were not identical. We investigated cell-specific effects of *ets-1*(RNAi) using fluorescence *in situ* hybridization (Fig. 4F). As with *in situ* hybridization, we saw significantly reduced cell number using individual glial markers (Fig. 4G,H). Looking at the overlap in gene expression, we saw that there were significantly fewer *estrella*^+^/*calamari*^+^ and *estrella*^−^/*calamari*^+^ cells in regenerating heads after *ets-1*(RNAi) (Fig. 4I,K). However, there was no significant difference in rare *estrella*^+^/*calamari*^−^ cells (Fig. 4J). Our data suggest that, in addition to impacting glial numbers overall, Ets-1 also influences gene expression in remaining glia, potentially reflecting additional roles in cell state or maturation. Overall, our data demonstrate a requirement for *ets-1* in glial cell maintenance, regeneration and gene expression in planarians.

### *ets-1* affects multiple *cathepsin*^+^ cell types

Continuous knockdown of *ets-1* eventually leads to animal death (Fig. S5) (Dubey et al., 2022). This led us to ask how *ets-1* affects cell types beyond glia and pigment cells (Dubey et al., 2022; He et al., 2017). Single cell RNA sequencing (scRNA-seq) atlases cluster planarian pigment cells and glial cells with other cells that express *cathepsin* (Fincher et al., 2018; Plass et al., 2018) (Fig. S6A). We first confirmed previous reports that *ets-1* affects pigment cells in planarians (Fincher et al., 2018; He et al., 2017; Stubenhaus et al., 2016) (Fig. S6B). After *ets-1*(RNAi), relative gene expression of pigment markers *pgbd-1* and *gst* was reduced by 12% and 28% relative to controls (Fig. S6B).

We also examined transcript levels for several genes expressed broadly in *cathepsin^+^* cells – *forkhead box factor-1* (*foxf-1*) (Scimone et al., 2018), *cathepsin F* (*ctsf*) and *low density lipoprotein receptor related-3* (*ldlrr-3*) (Roberts-Galbraith et al., 2016). Compared with control, *ets-1*(RNAi) animals had significant reduction across all three *cathepsin^+^* cell markers (Fig. S6C). The *cathepsin^+^* cluster (also known as the parenchymal cluster) also includes eight cellular subclasses or states that are uncharacterized, many of which express *ets-1* (Fincher et al., 2018; Plass et al., 2018) (Fig. S6A). We repeated RT-qPCR using eight additional genes that mark individual *cathepsin^+^* subclusters. After *ets-1*(RNAi), expression of these genes was significantly reduced (*aqp1*, *dd_5690* and *dd_9*), significantly increased (*dd_1831*) or unchanged [*TTPA*, *dd_7593*, *cathepsin L2* (*ctsl2*) and *protein tyrosine phosphate receptor type* (*ptprt*)] (Fig. S6D,E). Our RT-qPCR data paint a complex picture of the roles of *ets-1* in *cathepsin^+^* cell types other than pigment cells and glia, but we do not see universal downregulation of transcripts that fits neatly with the conclusion that *ets-1*(RNAi) affects all cells in the *cathepsin^+^* cluster in the same way. We also re-analyzed previously published RNA-seq data on *ets-1*(RNAi) animals that used different RNAi and amputation paradigms (Dubey et al., 2022). We did not see consistent significant downregulation of genes enriched in *cathepsin^+^* cell subtypes that would indicate a uniform loss of some or all of these cells (Fig. S6F, Table S4).

We conclude that *ets-1* knockdown induces differences in gene expression across multiple *cathepsin^+^* subclusters that depends on the specific target gene, cell subpopulation and amputation site chosen. Although our data strongly implicate *ets-1* in maintenance and regeneration of planarian glia and confirm the role of *ets-1* in pigment cells, roles for *ets-1* in regulating other specific subclusters of *cathepsin*^+^ cell types will require further detailed study. Importantly, glial cells are the only *cathepsin^+^* cell type present specifically within the nervous system, which allowed us to use *ets-1(RNAi)* as a first step for perturbing glia and examining neuronal organization and animal behavior.

### Reduction of *ets-1* affects CNS neuron gene expression and organization

Across metazoans, glial cells have been extensively implicated in neuronal development and physiology. For example, *Drosophila* glia regulate neuronal proliferation and, consequently, neuronal numbers (Coutinho-Budd et al., 2017; Ebens et al., 1993; Plazaola–Sasieta et al., 2019). Glial cells in *C. elegans* demarcate regions within the nervous system (i.e. nerve ring formation) before neuronal migration and, consequently, also regulate neuron numbers (Rapti et al., 2017; Yoshimura et al., 2008). We showed that *ets-1*(RNAi) affects planarian glial gene expression and glial cell number. This discovery allowed us to investigate potential consequences of glial cell perturbation in planarians.

**Fig. 5.**
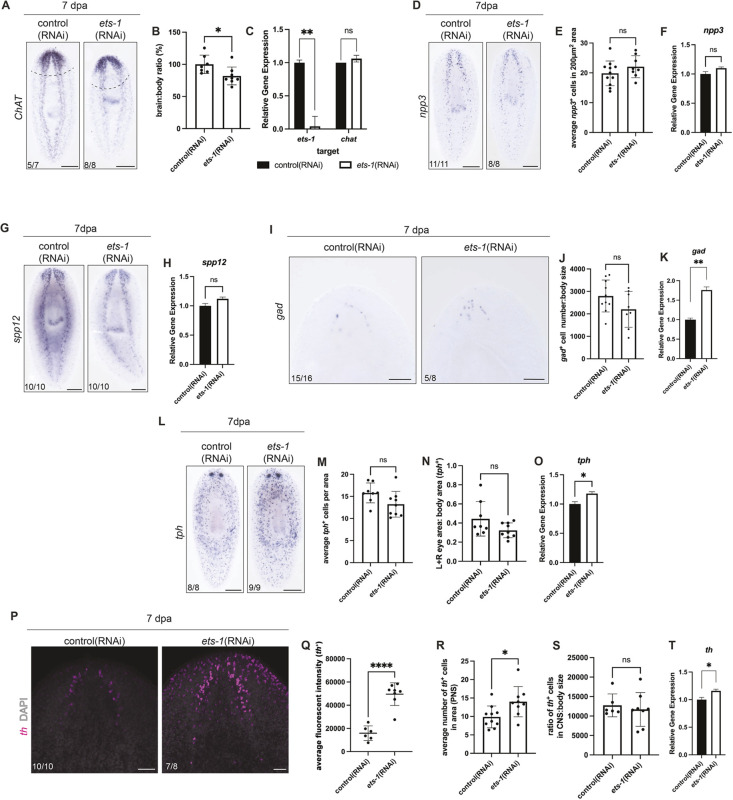
**Knockdown of *ets-1* does not affect neuronal cell numbers.** (A) *ChAT in situ* hybridization of regenerated control and *ets-1*(RNAi) animals*.* Dashed line indicates amputation site. (B) Brain-to-body ratio quantification shows reduced brain area for *ets-1*(RNAi) animals. Unpaired *t*-test with Welch's correction. Data are mean±s.d. (C) RT-qPCR was used to detect levels of *ets-1* and *ChAT* transcripts in regenerated RNAi animals; the same cDNA samples were used for Fig. 5F,H,K,O,T. Unpaired *t*-test. Data are mean±s.e.m. (D) 7 dpa control and *ets-1*(RNAi) animals subjected to *in situ* hybridization for *neuropeptide precursor*-3 (*npp3*). (E) *npp-3*^+^ cells counted in 200 µm^2^ areas throughout the body. Unpaired *t*-test with Welch's correction. Data are mean±s.d. (F) *npp-3* transcript levels detected with RT-qPCR in regenerated RNAi animals. Unpaired *t*-test. Data are mean±s.e.m. (G) *In situ* hybridization of 7 dpa control and *ets-1*(RNAi) animals showing *secreted peptide prohorome*-12 (*spp12*). (H) *spp-12* transcript levels detected with RT-qPCR in regenerated RNAi animals. Unpaired *t*-test. Data are mean±s.e.m. (I) *In situ* hybridization of control and *ets-1*(RNAi) animals showing *glutamic acid decarboxylase* (*gad*)*.* 62.5% of *ets-1*(RNAi) animals had disorganized *gad*^+^ arch pattern. (J) Quantification of *gad*^+^ cells, normalized to body size. Unpaired *t*-test with Welch's correction. Data are mean±s.d. (K) *gad* transcripts levels detected with RT-qPCR in regenerated RNAi animals. Unpaired *t*-test. Data are mean±s.e.m. (L) *In situ* hybridization of regenerated animals in control and *ets-1*(RNAi) animals with *tryptophan hydroxylase* (*tph*). (M,N) Quantification of *tph*^+^ cells in specific areas throughout the body and within the eye compared with body size. Unpaired *t*-test with Welch's correction. Data are mean±s.d. (O) RT-qPCR detecting *tph* transcript levels in regenerated RNAi animals. Unpaired *t*-test. Data are mean±s.e.m. (P) 7 dpa control and *ets-1*(RNAi) animals subjected to fluorescence *in situ* hybridization for *tyrosine hydroxylase* (*th,* magenta) and stained with DAPI (gray). (Q) Average intensity of *th* fluorescence *in situ* hybridization was quantified for control and *ets-1*(RNAi) animals. Unpaired *t*-test with Welch's correction. Data are mean±s.d. (R) *th*^+^ cells in the PNS were counted in 100 µm^2^ areas. Unpaired *t*-test with Welch's correction. Data are mean±s.d. (S) Quantification of *th*^+^ cells in the CNS was quantified in control and *ets-1*(RNAi) animals, and normalized to body size. Unpaired *t*-test with Welch's correction. Data are mean±s.d. (T) RT-qPCR detecting *th* transcript levels in regenerated RNAi animals. Unpaired *t*-test. Data are mean±s.e.m. *P≤0.05, **P≤0.01, ****P≤0.0001; ns, not significant. Scale bars: 200 µm in A,D,G,L; 100 µm in I; 50 µm in P.

We first asked whether perturbation of *ets-1* impacted gross morphology of the nervous system. We knocked down *ets-1* and examined expression of *ChAT* at 7 dpa (Nishimura et al., 2010) (Fig. 5A). We quantified brain area relative to body area and saw a significant decrease in brain size after *ets-1*(RNAi), with no change to *ChAT* transcript levels (Fig. 5B,C). Next, we examined whether specific neuronal subtypes were affected, coincident with the loss of glia. We performed *in situ* hybridization on *ets-1*(RNAi) animals after regeneration and examined expression of neuronal markers *neuropeptide precursor-3* (*npp-3*; Collins et al., 2010), *secreted peptide prohormone -12* (*spp12;* Ong et al., 2016; Shimoyama et al., 2016), *glutamic acid decarboxylase* (*gad*; Nishimura et al., 2008b), *tryptophan hydroxylase* (*tph*; Nishimura et al., 2007b), *tyrosine hydroxylase* (*th*; Fraguas et al., 2012; Nishimura et al., 2007a), *tyramine beta-hydroxylase* (*tbh;* Nishimura et al., 2008a) and *cintillo* (Oviedo et al., 2003) (Fig. 5D-T; Fig. S7A-D). Cell numbers for each neuronal cell type in the CNS were unaffected by *ets-1*(RNAi) (Fig. 5D,E,I,J,L-N,P,R,S; Fig. S7B,D). Interestingly, we noted that 62.5% of the regenerated *ets-1*(RNAi) animals had abnormal patterning of *gad*^+^ cells, in which the linear arched organization of *gad*^+^ cells seen in control animals was lost in *ets-1*(RNAi) animals (Fig. 5I). In all, we saw only a small increase in the number of *th*^+^ cells in the PNS in *ets-1*(RNAi) animals (Fig. 5R). Furthermore, we saw no changes to neuronal cell numbers or organization in uninjured animals after *ets-1* perturbation (Fig. S7E-K).

Despite no overt change in neuronal number, we observed that regenerated *ets-1*(RNAi) animals had significantly increased mRNA levels for *gad* and, to a lesser extent, *th* and *tph* (Fig. 5K,O,T). We also observed a significant increase in *th* expression within individual cells after *ets-1*(RNAi) (Fig. 5P,Q), which could explain the increased global *th* transcript levels. Other neuronal transcripts, including neuropeptide-encoding mRNAs *npp-3* and *spp-12* were not affected by *ets-1*(RNAi) (Fig. 5F,H). Based on our data, we conclude that loss of *ets-1* impacts several neuronal mRNAs but perturbs cell number for only rare subsets of neurons (e.g. PNS *TH+* cells) after regeneration.

Taken together, our data show that the reduction of *ets-1* results in: decreased brain size, altered organization of specific cell types (*gad*), anomalous neuronal gene expression and no change in cell number for neuron types of the brain. After the reduction in glial numbers, we did not see phenotypes like paralysis or death that would indicate widespread loss of neuronal function or failure to specify neurons during regeneration. Our data together indicate that inhibition of *ets-1*, likely through loss of glial cells, impacts planarian neuronal gene expression and CNS organization during head regeneration.

### Loss of *ets-1* leads to changes in neural connectivity

**Fig. 6.**
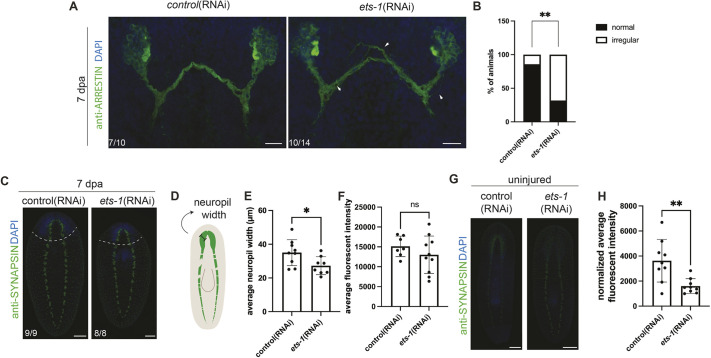
***ets-1* knockdown results in changes in neural architecture.** (A) 7 dpa regenerated control and *ets-1*(RNAi) animals were subjected to immunofluorescence against arrestin (photoreceptor axons, green) and stained with DAPI (blue). *ets-1*(RNAi) animals exhibited several defects in axon fasciculation (arrowheads). (B) Percentage of control and *ets-1*(RNAi) animals exhibiting one or more defects in axon fasciculation. See Materials and Methods for our criteria for ‘irregular’ organization. Fisher's exact test. (C) Immunofluorescence for anti-synapsin (synapses, green) and DAPI staining (nuclei, blue) in regenerated control and *ets-1*(RNAi) animals. Dashed line indicates amputation site. (D) Illustration of neuropil width measurement. (E,F) Quantification of neuropil width (E) and average fluorescence intensity (F). Unpaired *t*-test with Welch's correction. (G) Immunofluorescence for anti-synapsin (green) and DAPI staining (nuclei, blue) in uninjured control and *ets-1*(RNAi) animals. (H) Quantification of average fluorescence intensity. Unpaired *t*-test with Welch's correction. *P≤0.05, **P≤0.01; ns, not significant. Data are mean±s.d. Scale bars: 20 µm in A; 100 µm in C; 200 µm in G.

Glia also play important roles in axon guidance, axon fasciculation and axonal targeting in other species (Hidalgo et al., 1995; Mason and Sretavan, 1997; Poeck et al., 2001). In planarians, individual axon trajectories can be most clearly seen in the visual system. Furthermore, we noted that *estrella* can be seen in cells near the eyespots (Fig. 3G) (Roberts-Galbraith et al., 2016). To determine whether glial cells play roles in photoreceptor axon trajectory in planarians, we performed *ets-1*(RNAi) to reduce glial cell number and stained using anti*-*arrestin antibodies (Sakai et al., 2000) (Fig. 6A). We examined *ets-1*(RNAi) animals for defects in axon fasciculation (i.e. stray bundles or axons near optic chiasma or photoreceptor neurons, gaps in the optic chiasma) compared with controls (Fig. 6B). We found that *ets-1*(RNAi) animals had significantly increased rates of these defects when all criteria were considered together (Fig. 6A,B). When considered individually, each category of defect was enriched in *ets-1*(RNAi) animals by nonsignificant margins (Fig. S8A-D). We concluded that *ets-1* subtly affects axonal organization in the photoreceptor system.

Glial cells in other animal species play important roles in synapse organization and function (Eroglu and Barres, 2010; Lee and Chung, 2019; Neniskyte and Gross, 2017). As described, we observed decreased brain size after *ets-1*(RNAi) without a decrease in neuronal cell numbers (Fig. 5). We hypothesized that the decrease was due to the changes in the neuropil, a synapse- and process-rich structure in the interior of the planarian brain that is the primary location of most CNS glial cells. To determine whether *ets-1* knockdown affected synapse density or organization in planarians, we stained 7 dpa animals using anti-Synapsin antibodies (Fig. 6C) (Ross et al., 2015). We quantified medial brain gap (the space between the two brain lobes), average VNC gap length, average gap sizes, average width of the neuropil and average fluorescence intensity (Fig. 6D-F, Fig. S8E-H). Regenerated *ets-1*(RNAi) worms had a significantly decreased neuropil width compared with controls (Fig. 6E). However, we saw no additional effects on organization or staining intensity in the synapses of regenerated *ets-1*(RNAi) worms (Fig. 6C,F, Fig. S8E-H). Reduction in neuropil size could explain our previous observation of a small brain size after *ets-1*(RNAi) without a decrease in neuronal cell number (Fig. 6B). We also asked whether synapse density or organization was affected in uninjured animals (Fig. 6G,H). Interestingly, we observed significantly decreased average fluorescence intensity and increased neuropil width in intact *ets-1*(RNAi) animals compared with control (Fig. S8I-N), suggesting that *ets-1*(RNAi) also affects maintenance of synaptic density. Taken together, our data suggest that perturbation of *ets-1*, possibly through loss of glial cells, results in defects in axon and synaptic organization during homeostasis and regeneration.

### Perturbation of *ets-1* results in locomotion defects

**Fig. 7.**
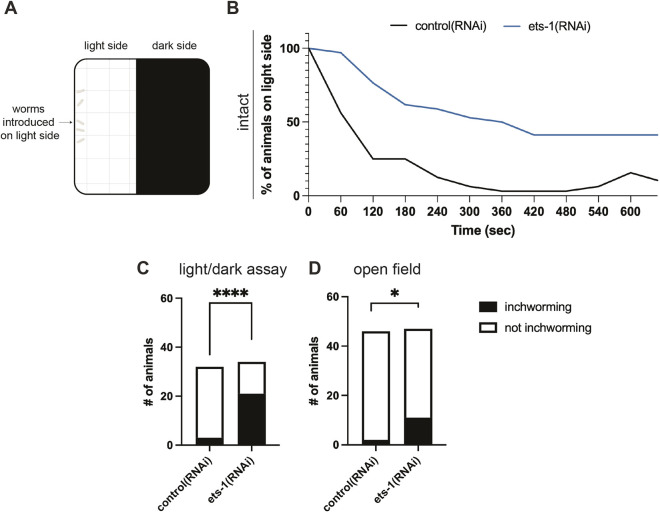
***ets-1* knockdown leads to changes in planarian behavior.** (A) Illustration of light/dark assay (Paskin et al., 2014; Zewde et al., 2018). (B) Graph shows percentage of intact animals on the light side (*n*=10-12 animals per replicate; three replicates, see also Fig. S9A). (C) Quantification of animals that exhibit inch-worming in the context of a light/dark assay. Fisher's exact test. (D) In an open field, *ets-1*(RNAi) still led to a significantly higher incidence of inch-worming behavior. Fisher's exact test. *P≤0.05, ****P≤0.0001.

We reasoned that reduction in glial cell number might, through the changes that we have detailed in previous sections, affect neuronal function. Impaired neuronal function would be reflected in planarian behavior, including response to light. Planarians normally exhibit negative phototactic behavior, preferring to move toward areas of low light (Fig. 7A, Fig. S9, Movie 1A,B; based on assays by Paskin et al., 2014; Zewde et al., 2018). *ets-1*(RNAi) animals consistently showed reduced ability to move into a dark space compared with control animals (Fig. 7A,B, Movie 1A,B). By 5 min, more than 50% of *ets-1*(RNAi) animals remained on the light side of the dish and by 10 min, 41.18% of *ets-1*(RNAi) animals failed to reach the dark side (Fig. 7B; Movie 1B). In comparison, 75% of control worms reached the dark side within 3 min; by 6 min, over 96% of control worms had reached the dark side (Fig. 7B, Fig. S9, Movie 1A). Interestingly, we noticed that *ets-1*(RNAi) worms often exhibited uncoordinated movements that included head lifts and inch-worming – a slow locomotion gait based on muscle contraction instead of cilia-mediated movement (Fig. S9C, Movie 1B). When we quantified inch-worming behavior, 61.76% of *ets-1*(RNAi) animals exhibited this behavior as they initiated movement compared with control animals (9.38%) (Fig. 7C). Even within an open field, 23.4% of *ets-1*(RNAi) animals exhibited inch-worming behavior at movement onset compared with 4.35% of control animals (Fig. 7D, Movies 2A,B). We verified that photoreceptors, ciliated *soxb1-2*^+^ neuronal numbers and other neuronal numbers relevant to photophobic movement were unchanged in *ets-1*(RNAi) animals (Fig. S9D-J). Therefore, we conclude that *ets-1*(RNAi) impacts both the quality and outcome of planarian movement, likely through glial roles in robust neuronal function or connectivity.

## DISCUSSION

Planarians regenerate their nervous systems quickly and with high fidelity, making them an attractive model for studying glia during successful nervous system repair. In this work, we established timelines for development and regeneration of glial cells, demonstrating that glia arise after neurons and depend on neurons for their regenerative placement throughout the nervous system. We further show that transcription factor *ets-1* plays conserved roles in gliogenesis and glial maintenance in *Schmidtea mediterranea*. Our work further leveraged our findings with *ets-1* to explore potential roles of glial cells in planarians for the first time. Our results indicate that *ets-1*(RNAi) causes altered gene expression in neuronal cell types, reduction in neuropil volume and perturbed fluidity of animal movement. It is important to note that *ets-1* affects multiple *cathepsin*^+^ cell types in planarians. Further work will be required to dissect the roles of Ets-1 in diverse cell types and to ablate glia more specifically to confirm and expand possible role(s) for glia in regulating neural physiology and behavior. Moreover, it will be interesting to address how *ets-1* specifies glial fate and to explore the identity and function of Ets-1 targets in planarian glial cells.

### The role of *ets-1* in gliogenesis is conserved in planarians

Members of the *Ets* family have conserved roles in driving gliogenesis across metazoans. ETS transcription factors are important for both CNS and PNS gliogenesis (Hagedorn et al., 2000). *Drosophila* Pointed, an ETS transcription factor expressed in glial cells, is required for differentiation of longitudinal and midline glial cells (Klaes et al., 1994; Klämbt, 1993). *Ets-1* is expressed in human cortex astrocytes and plays roles in astrocyte differentiation, proliferation and regulation of genes involved in astrocyte signaling (Amouyel et al., 1988; Fleischman et al., 1995). ETS family transcription factors are also crucial for survival and proper regeneration of mammalian Schwann cells and zebrafish bridging glia after injury (Arthur-Farraj et al., 2012; Klatt Shaw et al., 2021; Nagarajan et al., 2002; Parkinson et al., 2002). Similarly, we found that planarian *ets-1* is required for glial regeneration and maintenance. *ets-1* knockdown reduces glial numbers, with distinct effects on individual glial markers.

Planarian *ets-1* also affects pigment cells (He et al., 2017) and we showed that it regulates gene expression in other cell types in the planarian body, most of which are not well characterized. Interestingly, planarian glial cells cluster more closely with pigment cells than neurons in single-cell transcriptomic analyses (Fincher et al., 2018). This finding argues against common progenitors for neurons and glia in planarians, and suggests that glia might share a common progenitor with other phagocytic, *cathepsin^+^* cell types (e.g. pigment cells). Alternatively, *ets-1* could regulate cell state in a wide variety of phagocytic *cathepsin^+^* cell types that are not lineage related but cluster together due to similarities in gene expression that correspond to functional rather than lineage relationships.

### Relationships between planarian glia and neurons

During CNS development in vertebrates and *Drosophila*, neurogenesis often precedes gliogenesis as stem cells produce neurons before switching programs to make glia (Campbell and Götz, 2002; Miller and Gauthier, 2007; Molofsky and Deneen, 2015). This sequence of events is shared across species with varying time scales. Here, we uncovered a similar ‘birth order’ in planarian regeneration and development, in which neurons arise first and glia arise later. Whereas vertebrates and *Drosophila* progenitors undergo a neurogenic-to-gliogenic switch during neural development, planarian neurons and glial cells are thought to arise from distinct lineages of pluripotent stem cells (Crews, 2019; Fincher et al., 2018; Miller and Gauthier, 2007; Plass et al., 2018; Roberts-Galbraith et al., 2016; Wang et al., 2016). Interestingly, although pluripotent stem cells give rise to all cells in the adult planarian body, this is the first observation to our knowledge of a ‘birth order’ of cell types that contribute to a common tissue in planarians. Our observations raise new questions regarding why and how planarian stem cells produce glia and neurons with different timing after injury.

### Potential roles for glial cells in the planarian nervous system

Glial cells fulfill diverse roles across animals, including regulating neuronal cell numbers and migration, aiding axon guidance and growth, promoting neuronal differentiation, regulating synapse formation and pruning, regulating ion homeostasis, providing metabolic support, participating in sensory systems, and helping and/or impeding response to injury (Allen and Lyons, 2018; Falk and Götz, 2017; Jäkel and Dimou, 2017; Oikonomou and Shaham, 2011; Ortega and Olivares-Bañuelos, 2020; Shaham, 2015; Yildirim et al., 2019).

Our studies with Ets-1 allowed us to investigate potential roles for planarian glia for the first time. Our data indicate that planarian glia promote brain organization. We observed disorganized *gad*^+^ cells after *ets-1* knockdown, suggesting that glial cells might play a role in proper patterning for specific neurons. In addition, we observed reduction of neuropil width after *ets-1*(RNAi) in regeneration and reduction in synapsin staining in the neuropil after *ets-1*(RNAi) in homeostasis. The neuropil is a process- and synapse-rich region devoid of neuronal cell bodies, and is the primary location of glial cells. Spatial localization of planarian glia suggests possible roles in assisting in axon and synapse maturity, organization or function. Further tools to study synaptic structure and activity (i.e. calcium signaling) will be essential for investigation of glial effects on neurotransmission and synapse function. Conversely, our data do not support broad roles for planarian glial cells in regulating neuronal numbers or neuronal survival.

Glial expression of *glutamine synthetase* and *excitatory amino acid transporter* leads us to hypothesize that planarian glial cells regulate neurotransmitter uptake and recycling (Roberts-Galbraith et al., 2016; Wang et al., 2016). We also note that when *ets-1* is perturbed and glial cells are reduced, there is an increase in expression of several genes that encode neurotransmitter biosynthesis enzymes with no corresponding increase in neuronal number (e.g. *gad*). One possible explanation for this observation is that neurons may alter gene expression in response to persistence of extracellular neurotransmitters in the absence of recycling by glial cells (Araque et al., 1999; Oliet et al., 2001).

Additionally, planarian glia are likely to exhibit phagocytic properties, based on their classification as *cathepsin*^+^ cells (Fincher et al., 2018) and functional analyses (Scimone et al., 2018). Vertebrate glia (notably astrocytes, microglia and Schwann cells), *Drosophila* glia and *C. elegans* glia phagocytose apoptotic neurons and neurite debris during development and after neuronal injury (Aldskogius and Kozlova, 1998; Jessen and Mirsky, 2016; Jung and Chung, 2018; Logan and Freeman, 2007; Sulston et al., 1983). However, new tool development will be necessary to assess the purpose of phagocytosis by planarian glia as well as additional roles for glia in modulating neurons.

### Planarian glial cells in behavior and beyond

Our data indicate that loss of glial cells is associated with changes in planarian behavior, based on our finding that *ets-1*(RNAi) impacts the quality and speed of movement in planarians. Locomotion defects were more pronounced when *ets-1*(RNAi) animals initiated movement. We know little about whether planarian glia may impact movement locally at the neuromuscular junction (NMJ), or whether glia impact integration of sensory information, decision making and initiation of movement through neurons in the CNS. In vertebrates, the NMJ is composed of a presynaptic motor neuron terminal, a post-synaptic muscle cell and perisynaptic glial cells (typically Schwann cells) that cooperate for motor output (Reddy et al., 2003). Further ultrastructural work could reveal whether planarian glia reside near synapses between neurons and/or between neurons and other cell types.

More work will also be required to determine the basis of movement defects in *ets-1*(RNAi) animals. One possibility is that dysregulation of neurotransmitter abundance in *ets-1*(RNAi) animals impacts negative phototaxis. In particular, *gad^+^* GABAergic neurons and *th^+^* dopaminergic neurons affect movement in planarians (Nishimura et al., 2007a,b, 2008a,b). Both *gad* and *th* mRNAs are dysregulated in *ets-1*(RNAi) animals. Further work will explore whether neurotransmitter levels are impacted in the absence of glia and whether exogenous neurotransmitters or antagonists could rescue behavioral defects in *ets-1*(RNAi) animals.

In conclusion, a thorough characterization of planarian glia fills gaps in our understanding of glia in an underexplored, highly regenerative phylum. Future work may reveal fascinating new aspects of glial biology, and provide insight into glial evolution, development and regeneration. Our work with planarian glia provides a valuable point of comparison and contrast with glial cells in other organisms, particularly in the areas of glial function and glial response to injury.

## MATERIALS AND METHODS

### Animal husbandry

A clonal line of diploid asexual *Schmidtea mediterranea* (CIW4 strain) was maintained at 18-22°C in the dark. Animals were kept in Ziploc (9 cup) reusable containers in 1×Montjuïc salts as previously described (Cebrià and Newmark, 2005). Organic puréed calf or beef liver (White Oak Pastures, GA, USA) was used to feed animals once per week. Animals were starved for a minimum of 1 week before use in experiments. For sexual *Schmidtea mediterranea* husbandry and embryo staging, outbred cohorts of sexually reproducing planarians descended from animals collected in Sardinia by Dr Maria Pala (in 1999) and Drs Longhua Guo and Alejandro Sánchez Alvarado (in 2015) were reared in 1×Montjuïc salts (Cebrià and Newmark, 2005) at 20°C in constant darkness. Sexually mature animals were housed at low density and fed homogenized beef liver (White Oak Pastures) twice per week. To promote fertility, breeding adult sexual *Schmidtea* stocks were replaced every 3 months with young adults (6-8 weeks post-hatching) or adult regenerates (6-8 weeks post-amputation). Egg capsules were collected daily, soaked in 10% bleach for 3 min, rinsed four to six times, and stored in 1×Montjuïc water in a 20°C incubator.

### Identification of genes and cloning

Planarian homologs of genes of interest were identified from PlanMine 3.0 (Rozanski et al., 2019) based on homology and from scRNA-seq data (Fincher et al., 2018). Primers shown in Table S1 were designed using Primer3 (Rozen and Skaletsky, 1999) to PCR-amplify 500-750 bp segments of genes of interest from asexual *S. mediterranea* cDNA synthesized with an iScript kit (Bio-Rad) (Collins et al., 2010). Each PCR product was ligated into Eam1105I-digested pJC53.2 vector for use in RNAi and *in situ* hybridization experiments using standard molecular biology protocols (Collins et al., 2010).

### Protein alignment and phylogenetic analysis

Protein alignment and phylogenetic analysis were performed as described previously (Jenkins and Roberts-Galbraith, 2023). Briefly, the longest open reading frame for each sequence was identified using web-based translation tool Expert Protein Analysis System [ExPASy, https://www.expasy.org/; (Gasteiger, 2003)] or NCBI Open Reading Frame Finder (https://www.ncbi.nlm.nih.gov/orffinder/). Protein sequences of Ets-1 from other species were aligned to reference sequences (Table S2). Phylogeny was performed at www.phylogeny.fr (Dereeper et al., 2008) using MUSCLE for sequence alignment with the ‘A la Carte’ option (Edgar, 2004) and PhyML for phylogenetic tree construction (Guindon et al., 2010).

### RNAi experiments

For RNAi experiments, animals (10-12 worms; 3-4 mm in size) were kept in 60 mm Petri dishes, washed after feeding and supplemented with 1:1000 gentamicin sulfate (50 mg/ml stock, Gemini Bio-Products) throughout the experiment. dsRNA was synthesized using standard molecular biology techniques (Collins et al., 2010). dsRNA matching *Aequorea victoria green fluorescent protein* (*GFP*) was used for negative control feedings. For *ets-1*(RNAi) paradigms, animals were fed 5-10 µg dsRNA mixed with 25-30 µl of 3:1 beef liver:1×Montjüic salts mixture and 2 µl McCormick green food dye. Feedings were completed every 3 days for a total of five or six feedings. For regeneration experiments, animals were amputated pre-pharyngeally 7 days after the last feeding and fixed according to different downstream protocols. For *ovo*(RNAi) and *soxb1-2*(RNAi), animals were fed 5 µg dsRNA every 4-5 days for a total of four feedings. For *coe*(RNAi) and *sim*(RNAi), animals were fed 5 µg dsRNA every 3 days for five feedings. Amputation and fixation were performed as previously described. For *ndk*(RNAi) paradigms, animals were fed 5 µg dsRNA every 3 days for three feedings. Amputation was performed as before and animals were fixed 10 days after regeneration. For long-term *ets-1*(RNAi) experiments, 30 animals per RNAi condition were fed 5 µg or 10 µg dsRNA every 3 days for a period of 60 days. The number of surviving animals was quantified as detailed in Fig. S5. Survival curves were plotted using GraphPad Prism9.

### *In situ* hybridization and immunofluorescence

Animals used for *in situ* hybridization were treated with 7.5% N-acetyl-L-cysteine (NAC) in phosphate-buffered saline [PBS; 1.37 M NaCl, 27 mM KCl, 100 mM Na_2_HPO_4_ and 20 mM KH_2_PO_4_ (pH 7.4)], and fixed in 4% formaldehyde in PBSTx (PBS+0.3% Triton-X 100). Riboprobes for *in situ* hybridization on asexual planarians were generated using PCR-amplified products from pJC53.2 vectors with T7 primers (orientations provided in Table S1). Antisense probes were synthesized with digoxigenin-11-UTP (Roche) or fluorescein-12-UTP (Roche) using standard molecular protocols (Collins et al., 2010). *In situ* hybridization experiments were performed on asexual animals as previously described (King and Newmark, 2013). Some samples were processed in an Insitu Pro (Intavis) hybridization robot. Probes were detected using anti-digoxigenin with Fab fragments (Sigma-Aldrich) conjugated to alkaline phosphatase (Roche) for colorimetric *in situ* hybridization or horse-radish peroxidase (Roche) for fluorescence *in situ* hybridization. For colorimetric *in situ* hybridization, 5-bromo-4-chloro-3-indolyl phosphate [BCIP (Roche)] and nitro blue tetrazolium chloride [NBT (Roche)] in alkaline phosphatase (AP) buffer were used for signal development. Animals were mounted in 80% glycerol. Samples were imaged with an Axiocam 506 color camera mounted on Zeiss Axio Zoom V.16 microscope using ZEN 2.3 pro software.

For fluorescence *in situ* hybridization, probes were detected using anti-digoxigenin and anti-fluorescein Fab fragments conjugated to horseradish peroxidase (Roche). 1:500 fluor-tyramide (TAMRA or FAM), 1:1000 4-IPBA and 0.003% H_2_O_2_ in Tyramide Signal Amplification (TSA) buffer [2 M NaCl and 0.1 M boric acid (pH 8.5)] was used for signal development for 45 min. Double fluorescence *in situ* hybridization samples were incubated in sodium azide solution [100 mM sodium azide (ThermoFisher Scientific) in PBSTx] for 45 min to inactivate peroxidase before secondary signal development. Fluorescence *in situ* hybridization samples were mounted in VectaShield Antifade Mounting Medium and imaged using a Zeiss LSM 880 confocal microscope with an upright AXIO Imager Z2 and ZEN Black 2.3 SPI software. Whole animals were imaged using a Plan-Neofluar 10×/0.3 objective and smaller fields were imaged with Plan-Apochromat 20×/0.8 objective (no immersion). For each channel, the cut-off between signal and background was determined using the fluorescence intensity range indicator. Images shown are representative.

Embryo staging was performed according to guidelines set forward previously (Davies et al., 2017). The collection date was considered to be 1 day post-egg capsule deposition (dped). Early spherical stage 6 (S6) embryos were fixed at 6-7 dped; elongating mid-stage 6 (mid-S6) embryos were fixed at 8 dped; stage 7 (S7) embryos were fixed at 10 dped; stage 7.5 (S7.5) embryos were fixed at 12 dped; and stage 8 (S8) embryos were fixed at 14-15 dped using a protocol described previously (Davies et al., 2017) for colorimetric *in situ* hybridization with the following modifications. Embryos were removed from egg capsules immediately before fixation. S6 and S6.5 embryos were fixed for 6 h to overnight in 4% paraformaldehyde in PBSTx (PBS+0.5% Triton X-100) at room temperature. S7, S7.5 and S8 embryos were treated with 5% NAC in PBS (S7, 2 min; S8, 4 min) immediately followed by fixation in 4% paraformaldehyde in PBSTx (PBS+0.5% Triton X-100) for 45 min (S8) or 2 h (S7 and S7.5) at room temperature. Colorimetric whole-mount *in situ* hybridization images were acquired on a Zeiss Axio Zoom V16 equipped with a Axiocam 305 color camera. For image processing, a polygonal lasso tool was used to extract images of embryos from the original TIFFs; these were transferred to a white background along with the scale bar. Brightness and contrast were adjusted on colorimetric images to facilitate visualization of the colorimetric *in situ* hybridization signal.

Briefly, for immunofluorescence experiments, asexual planarians were treated in 2% HCl, fixed for 15 min in 4% formaldehyde in PBSTx, and then bleached in 6% H_2_O_2_ in PBSTx overnight. Animals were blocked in 1% bovine serum albumin (BSA) in PBSTx overnight at 4°C. The primary antibodies used were anti-synapsin (1:100, 3C11 concentrate; Developmental Studies Hybridoma Bank) and anti-arrestin (1:1000, cat 016-arrestin-01, LagenLabs). Secondary antibodies were used at a dilution of 1:500 and 1:1000 (goat anti-mouse Alexa Fluor 488 and goat-anti-rabbit Alexa Fluor 488, respectively; Invitrogen) (Ross et al., 2015; Sakai et al., 2000). Samples were mounted in VectaShield Antifade Mounting Medium and imaged using a Zeiss LSM 880 confocal microscope with ZEN Black software. Whole animals were imaged using a 10×/0.3 objective; specific regions were imaged with a 20×/0.8 objective (no immersion).

### Analysis of RNA-sequencing data

Analysis of glial gene expression during early regeneration of asexual planarians and development of sexual planarians was performed using previously published RNA-sequencing data (Davies et al., 2017; Roberts-Galbraith et al., 2016) and plotted using GraphPad Prism9. Re-analysis of *ets-1*(RNAi) gene expression across different *cathepsin*^+^ subclusters was performed using previously published RNA-sequencing data (Dubey et al., 2022) and expression profiles provided in previous transcriptome data analysis (Fincher et al., 2018; Plass et al., 2018).

### Real-time quantitative PCR

Total RNA was isolated from RNAi-treated animals (7 dpa, whole worms) using Trizol (Invitrogen) and the manufacturer's protocol. 1 µg of RNA was reverse transcribed into cDNA using an iScript cDNA Synthesis Kit (BioRad) as per the manufacturer's protocol. RT-qPCR was completed using Applied Biosystems QuantStudio3 Real-Time PCR system and GoTaq qPCR Master Mix with SYBR Green (Promega). All primers used for real-time quantitative PCR (RT-qPCR) are shown in Table S3. All measurements were performed in biological and technical triplicates; RNAi and RNA purification were performed from three individual Petri dishes (10-12 worms each, biological triplicates) for each RNAi condition, and then three identical qPCR reactions were completed per sample/primer pair (technical triplicates). Overall transcript normalization was accomplished using *beta-tubulin* mRNA within each sample. Statistical analyses were performed using GraphPad Prism9; details of each statistical test are provided in the figure legends.

### Quantification of ventral nerve cord gaps, brain-to-body ratio, cell numbers and neural structures

#### Quantification of VNC gaps (*estrella* staining)

For quantification of ventral nerve cord (VNC) gaps in *estrella* expression, FIJI (Schindelin et al., 2012) was used to measure overall VNC length per animal by summing the length of the left and right VNC. The number of true gaps (defined by the absence of *estrella* expression in VNC) was counted manually across animal body length. The length of each true gap was summed per animal and averaged. A ratio of gap length to total VNC length per animal was then determined using the sum of all gap lengths relative to the VNC length.

#### Quantification of cell numbers

For quantification of *estrella*^+^ cells in *ets-1*(RNAi) head regenerates, cells were counted manually in the new blastema for each animal and averaged. For quantification of *estrella*^+^ cells in the trunk pieces in *ets-1*(RNAi), *estrella*^+^ cells were counted for five separate pre- and post-pharyngeal in 100 µm^2^ area (intact) or 200 µm^2^ areas (regenerated) per animal and averaged, as previously described (Stelman et al., 2021). Fluorescence *in situ* hybridization images were quantified in Bitplane IMARIS 9.9 (Oxford Instruments) using spots or colocalization modules for quantification of *estrella*^+^, *calamari*^+^, *estrella*^+^/*calamari*^+^, *estrella*^+^/*calamari*^−^, *estrella*^−^/*calamari*^+^, *gad*^+^ and *th*^+^ cells in *ets-1*(RNAi) animals; *estrella*^+^ and *pkd2L-1*/*pkd1L-2*^+^ cells in *soxb1-2(*RNAi) animals; and *estrella*^+^ in *coe*(RNAi), *sim*(RNAi), *ndk*(RNAi) animals. Cells were counted in the new head blastema or specified regions for each animal within 250 µm^2^ or 200 µm^2^ areas, respectively. For quantification of *estrella*^+^ cells in and near the eye, *z*-stack images were analyzed using ImarisViewer 9.9.1 (Oxford Instruments) and then we manually quantified the presence or absence of *estrella*^+^ cells adjacent to eyespots (left and right) based on three-dimensional placement for each animal per condition. For *in situ* hybridization samples, quantification of individual markers was carried out as follows: for quantification of *gad*^+^ cells from colorimetric *in situ* hybridization, cell numbers were manually counted in head blastemas; for quantification of *npp3*^+^ and *tph*^+^ cells in *ets-1*(RNAi) animals, cell counts were averaged for five non-overlapping 200 µm^2^ boxes or six non-overlapping 100 µm^2^ boxes, respectively; for quantification of *tbh*^+^, cell numbers were quantified in the brain lobe and VNC, and compared with body size; for quantification of *pkd*^+^ cells in *ets-1*(RNAi) animals, cells were quantified in 100 µm^2^ boxes (head) or averaged from three boxes of 200 µm^2^ area (trunk) in the medial stripe and marginal PNS regions.

#### Quantification of brain or eye size

Brain-to-body ratios were determined by tracing the *ChAT*^+^ expression in the brain using FIJI and comparing brain area to body area, as previously described (Roberts-Galbraith et al., 2016; Schindelin et al., 2012). In these experiments, we normalize ratios so that controls are set at 100%. Similarly, the area of *tph*^+^ or *foxQ2*/*myoVIIA*/*sans*^+^ expression in the eyes was traced using FIJI (summation of left and right eye area) and compared with body size.

#### Quantifications of neural structures

For quantification of neural structures in anti-synapsin and anti-arrestin immunofluorescence images, identities of samples were masked and randomized for quantification. Traits were quantified from maximum intensity projection images (area) or single planes (intensity). For anti-arrestin, samples were quantified for the number of stray bundles or axons near the photoreceptors, the number of stray bundles or axons in and/or near the optic chiasma, the number of stray bundles or axons near the neuropil and the number of gaps in axon trajectory. We set parameters to describe axonal organization as follows: if the gap number was less than four and the total number of stray projections was less than two and if fraying in midline was 0, then axonal organization was within the range of control(RNAi) animals and was considered ‘normal’. If one or multiple parameters were not satisfied for an animal, it was counted as an irregular phenotype. For anti-synapsin, samples were quantified for neuropil width (brightest parts and including fainter edges), brain gaps and VNC gaps using FIJI. Brain width and brain gap criteria were measured in triplicate and averaged for each individual animal. For quantification of VNC gaps, the number of gaps on the left and right side was averaged, and then divided by the total length of the animal (in mm) to determine the average gap length per mm. To calculate the average gap length, measurements of each gap per individual sample were taken and then averaged per animal. The samples were then unmasked and measurements were averaged for each RNAi condition. All statistical analyses were conducted using GraphPad Prism9; details of each statistical test are shown in the figure legends.

### Behavioral assays

Before light/dark behavioral assays, RNAi animals were placed in VWR Square Petri Dishes (Electron Microscopy Sciences, 13×13 mm) after the last dsRNA feeding to acclimate to the dishes for 2 days. For the light/dark assay, new Petri dishes were half covered with black electrical tape (Duck Brand) on the lid and the corresponding side of the petri dish. Ten to 12 animals per RNAi condition were then introduced at the edge or corner of the light side of the Petri dish and recorded on an iPhone 11 (Apple) from a height of ∼17 cm from the top of the dish for ∼10 min. The behavioral test was repeated in biological triplicate for each experimental condition. To quantify, the number of animals remaining on the light side was recorded at 60 s intervals. 0 s indicates when animals were introduced to the dish. The percentage of animals (over three biological replicates) for each RNAi condition residing in the light side was then calculated for each time point and plotted using GraphPad Prism9. For publication, videos were sped to 20× using Adobe Photoshop; stills from videos were taken in 10 s increments using iMovie.

## Supplementary Material

10.1242/develop.201666_sup1 Supplementary information
