# Supplementary material for: Ets-1 transcription factor regulates glial cell regeneration and function in planarians
Source: Development. 2023 Sep 14;150(18):dev201666. doi: 10.1242/dev.201666 (PMC10508700; doi:10.1242/dev.201666)
Supplement: Supplementary information [file develop-150-201666-s1.pdf]

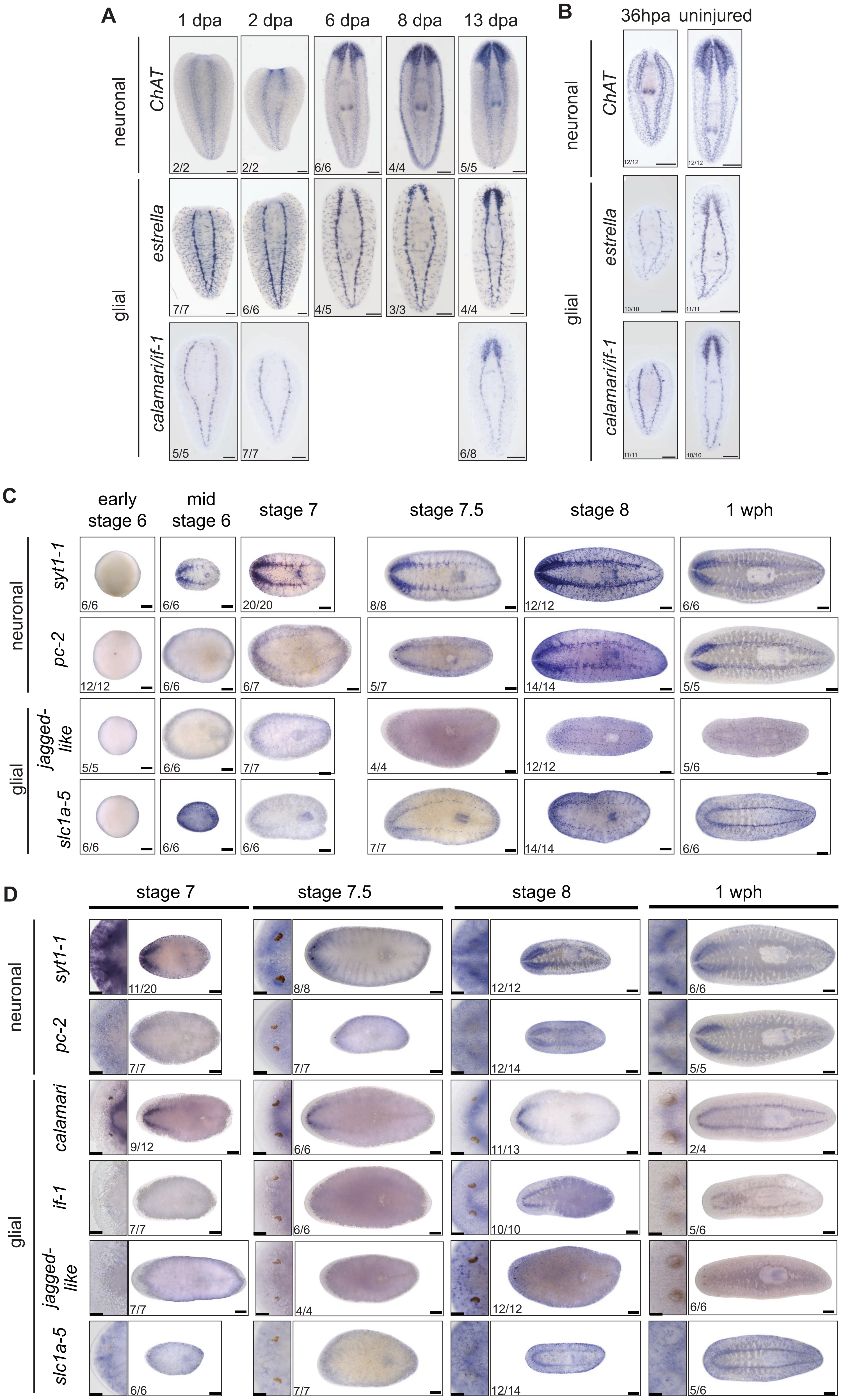

Development • Supplementary information

Fig. S1. Neuronal and glial gene expression during head regeneration.

(A) ISH regeneration timeline of neurons (*ChAT*) and glia (*estrella*, pooled *if-1*/*calamari*) in asexual planarians at 1, 2, 6, 8, and 13 days after head amputation (dpa). Ventral view, anterior up, from same time series as Fig 1A. (B) ISH of *ChAT* and glial markers 36 hours post head amputation (hpa) and in uninjured asexual planarians. (C) ISH of neuronal markers (*pc-2, syt1-1*) and glial markers (*slc1a-5/EAAT, jagged-like*) in planarian embryos (early S6, mid-S6, S7, S7.5, S8) and juveniles (1 week post-hatching). Anterior to left, ventral views. (D) ISH of neuronal markers *(pc-2, syt1-1)* and glial markers (*if-1*, *calamari*, *jagged-like*, *slc1a-5/eaat*) at indicated developmental stages. Dorsal views. Insets show expression in or near the eyes. Scale bars: whole animal images 200 µm; eyes 50 µm.

Development • Supplementary information

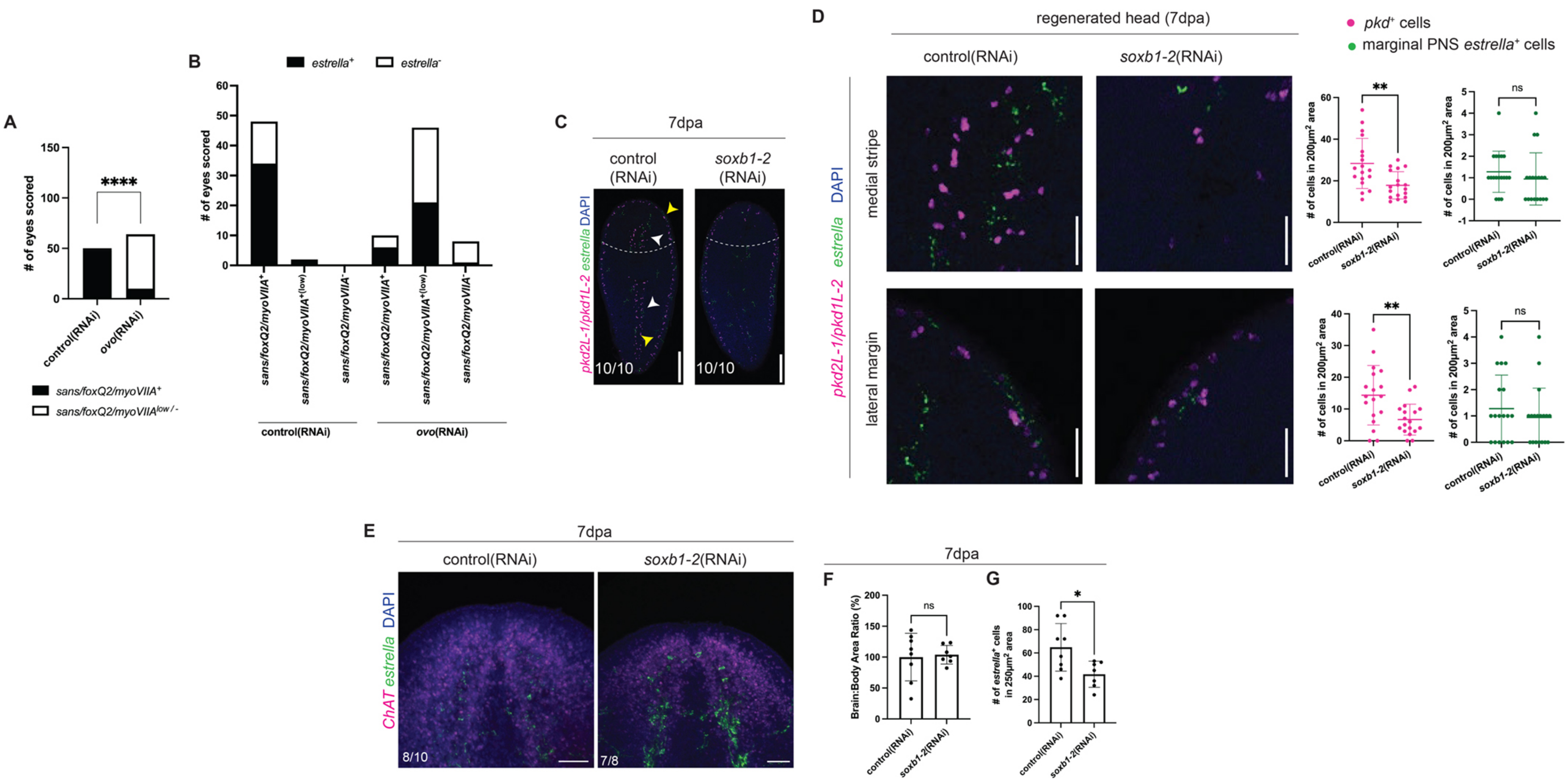

Fig. S2. Neural roles in glial regeneration.

(A) Quantification of photoreceptor neuron markers (pooled *sans*/*foxQ2*/*myoVIIA*) present (black) or reduced/absent (white) in control and *ovo*(RNAi) eyes. Left and right eyes were considered separately. Fisher's exact test. (B) Glia in *ovo*(RNAi) eyes separated by category based on *sans*/*foxQ2*/*myoVIIA* presence (+/low/-). (C) 7 dpa control and *soxB1-2*(RNAi) animals subjected to FISH detecting pooled *pkd2L-1*/*pkd1L- 2* (magenta), *estrella* (green), and DAPI (blue). Arrowheads indicate where dorsal *estrella*$^{+}$ cells were observed: medial stripe (white) and lateral margin (yellow). Dashed lines: amputation site. Whole body animal is shown from Figure 3I. Dorsal view. (D) Insets showing *estrella*$^{+}$ cells (green) and *pkd2L-1/1L-2*$^{+}$ cells (magenta) in regenerated head tissue in respective regions. Quantification of each cell type in given area is presented on the right. Unpaired t-test with Welch's correction. (E) 7 dpa control and *soxB1-2*(RNAi) animals subjected to FISH detecting *ChAT* (magenta) and *estrella* (green), and DAPI (blue). Unpaired t-test with Welch's correction. (F) Quantification of brain-to-body ratio for *soxb1-2*(RNAi) animals normalized to control. Unpaired t-test with Welch's correction. (G) Quantification of *estrella*$^{+}$ cells in regenerated heads in 200 μm^{2} areas of control and *soxb1-2*(RNAi) animals. Unpaired t-test with Welch's correction. Anterior up. *p-value≤0.05, **p-value≤0.01, ns=not significant. Scale bar: (C) 200 μm (D,E) 50 μm.

Development • Supplementary information

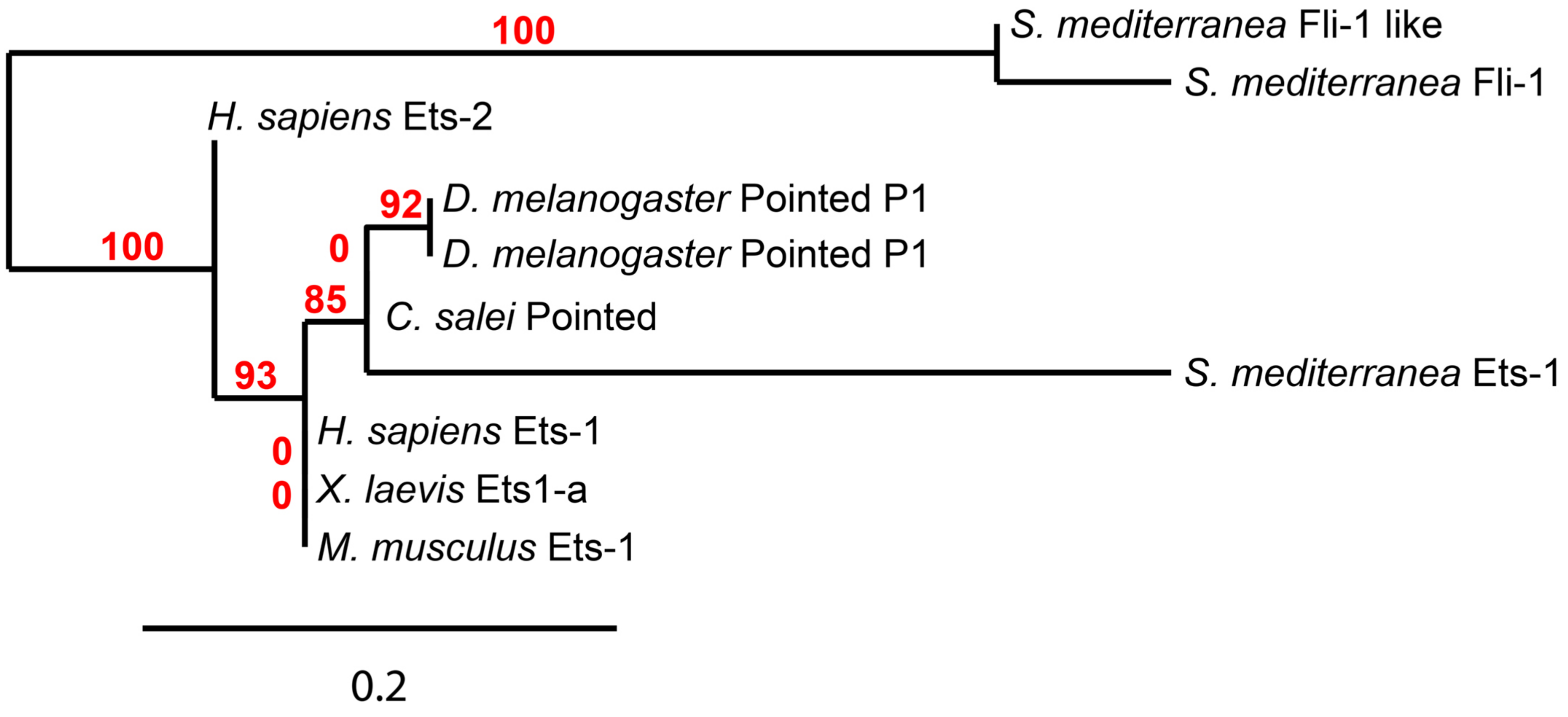

Fig. S3. Ets-1 protein sequence is highly conserved. Phylogenetic tree of Ets-1 protein sequence of 6 species based on longest open reading frame. Analysis shows the relationship between planarian Ets-1 and Ets-1 protein sequences in other species. The outgroup is planarian Fli-1 and Fli-1-like proteins, two proteins that possess an ETS domain, but have no established role in glial cells. Red text denotes the percentage of support for each node.

Development • Supplementary information

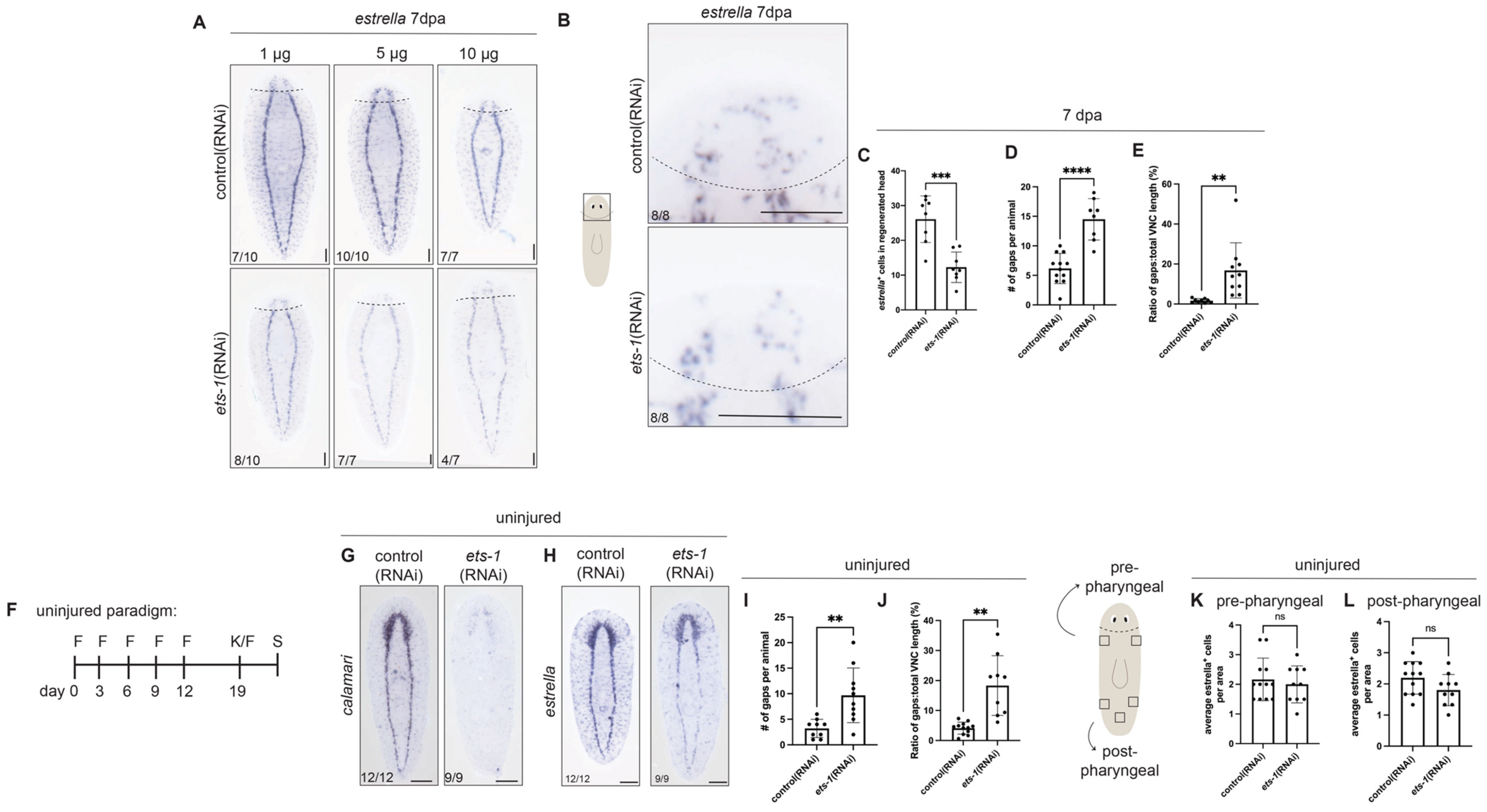

Fig. S4. Ets-1 knockdown phenotype on glial markers varies depending on dosage.

(A) Animals were fed 1 µg, 5 µg or 10 µg *in vitro*-transcribed dsRNA targeting *ets-1* for 5 feedings 3 days apart. Animals were amputated, fixed at 7 dpa, and subjected to ISH with *estrella*. 5 µg and 10 µg regimens yielded more robust reduction of *estrella*$^+$ cells in regenerated heads in *ets-1*(RNAi) animals. (B) *ets-1*(RNAi) resulted in fewer *estrella*$^+$ cells present in 7 dpa regenerated heads compared to control. (C) Quantification of *estrella*$^+$ cells in regenerated head blastemas in control and *ets-1*(RNAi) animals. Regenerated *ets-1*(RNAi) animals have (D) more gaps and (E) longer stretches of VNC gaps compared to control. Dashed lines: amputation site. (F) Uninjured RNAi paradigm. (G-H) Uninjured control and *ets-1*(RNAi) animals were subjected to ISH with glial markers *calamari* and *estrella*. (I-J) Quantification of number of gaps, and percentage of VNC with gaps in uninjured control and *ets-1*(RNAi) animals. (K-L) Quantification of *estrella*$^+$ cells in the PNS in uninjured control and *ets-1*(RNAi) animals. Unpaired T-test with Welch's correction. **p-value≤0.01, ***p-value≤0.001, ****p-value≤0.0001, ns=not significant (unpaired t-test with Welch's correction). Scale bar: 200 µm.

Development • Supplementary information

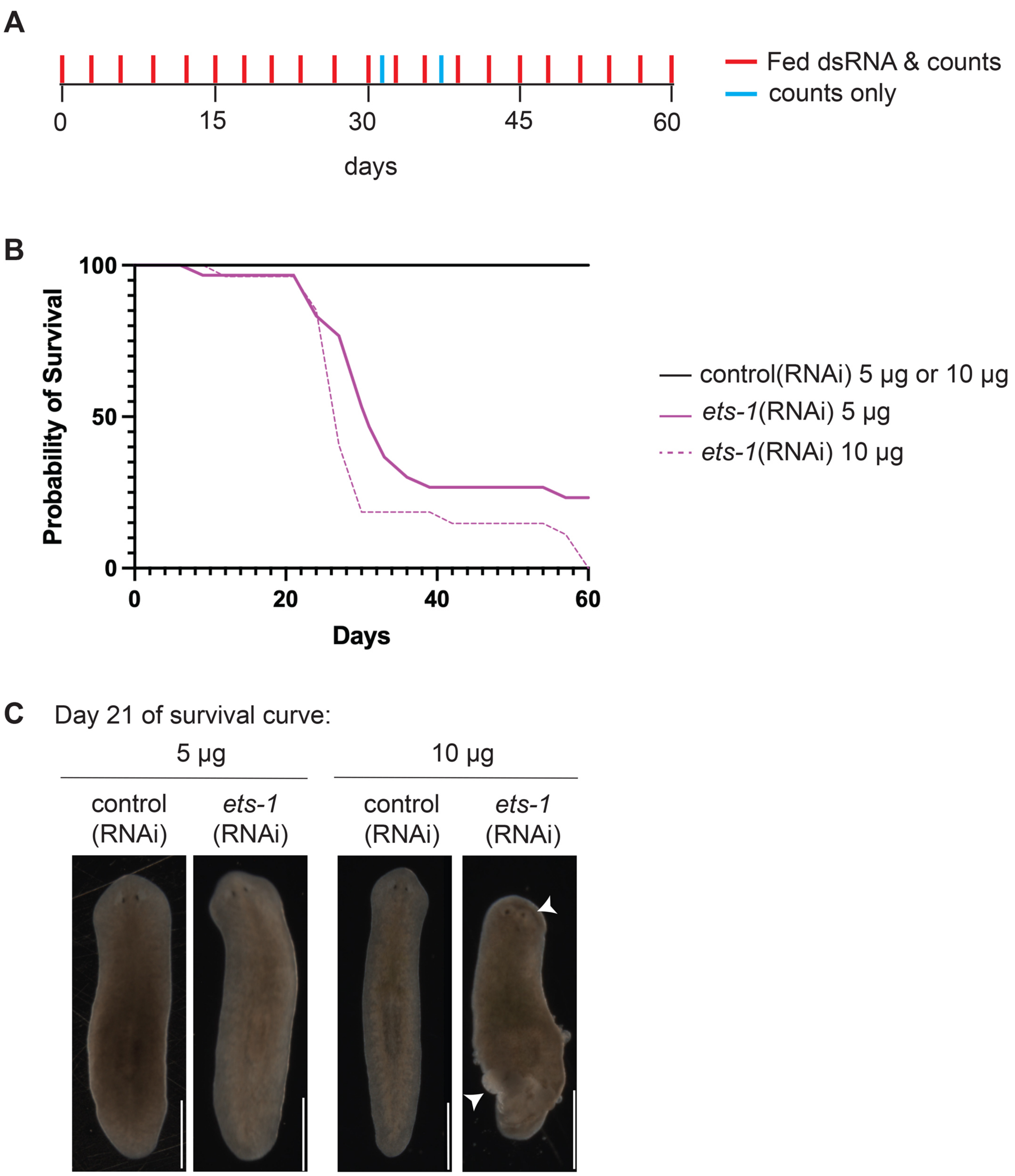

Fig. S5. *Ets-1* knockdown leads to eventual animal death.

(A-B) Animal cohorts were subjected to a 60-day dsRNA-feeding regimen, with feeding every 3 days. Red: Days with feeding and animal counts. Blue: days with animal counts only. Survival curve depicting the relative percentage of surviving animals at different dsRNA feeding doses after long term RNAi; N=30 animals each. (C) Live images of control and *ets-1*(RNAi) animals at day 21 (post 8th feeding). *ets-1*(RNAi) animals at 10 µg dsRNA exhibited lesions (white arrowheads) in the tail and around eyes that led to eventual lysis. Scale bar: 200 µm.

Development • Supplementary information

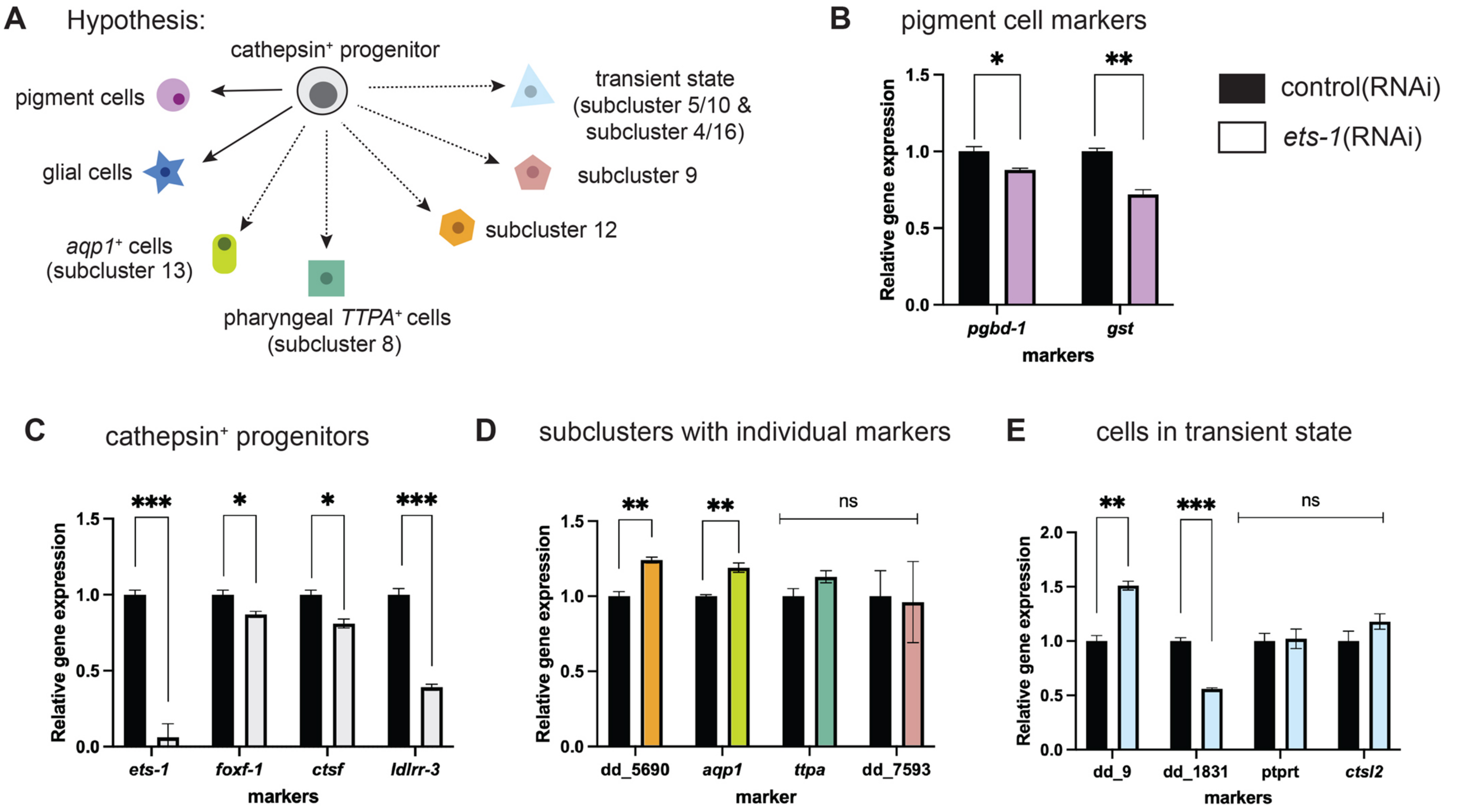

F

cell cluster	7 dpa (head) qPCR	5 dpa (sagittal) RNA-seq from Dubey *et al.* 2022	homeostasis RNA-seq from Dubey *et al.* 2022
○ cathepsin^{+} progenitor	↓	↓	↓
pigment cells	↓	↓↑	↑ (n.s.)
glial cells	↓	↓↑ (n.s.)	↓↑ (n.s.)
aqp1^{+} cells (subcluster 13)	↑	↓↑ (n.s.)	↓↑ (n.s.)
pharyngeal *TTPA*$^{+}$ cells (subcluster 8)	↑ (n.s.)	↓↑	↓↑
subcluster 12	↑	↓↑ (n.s.)	↓↑ (n.s.)
subcluster 9	↓ (n.s.)	↓	↓↑ (n.s.)
transient state (subcluster 5/10 & subcluster 4/16)	↓↑	↓	↓↑ (n.s.)

Fig. S6. *ets-1* affects gene expression in individual *cathepsin*$^{+}$ cell types in distinct ways.

(A) Graphical illustration of *cathepsin*$^{+}$ cell subclusters identified in single cell transcriptomic atlases (Fincher *et al.*, 2018; Plass *et al.*, 2018). Clusters are color-coded as follows: grey = *cathepsin*$^{+}$ progenitor cells, purple = pigment cells, dark blue = glial cells, light green = *aqp1*$^{+}$ cells, green = *TTPA*$^{+}$ cells, orange = subcluster 12, pink = subcluster 9, light blue = subclusters 5/10 and subcluster 4/16. Lineage relationships are drawn as currently hypothesized. (B-E) RT-qPCR was used to detect levels of *ets-1* and other markers of *cathepsin*$^{+}$ subclusters after RNAi with color coding as in A. Details of each marker are provided in Table S3. *p≤0.05, **p-value≤0.01, ***p-value≤0.001, ****p-value≤0.0001, ns=not significant (Unpaired t-test, error bars: SEM). (F) Summary table of trends seen after *ets-1*(RNAi) in each *cathepsin*$^{+}$ cell type from qPCR data and published RNA-seq data (Dubey *et al.*, 2022). Details of each gene analyzed under each subcluster are provided in Table S4.

Development • Supplementary information

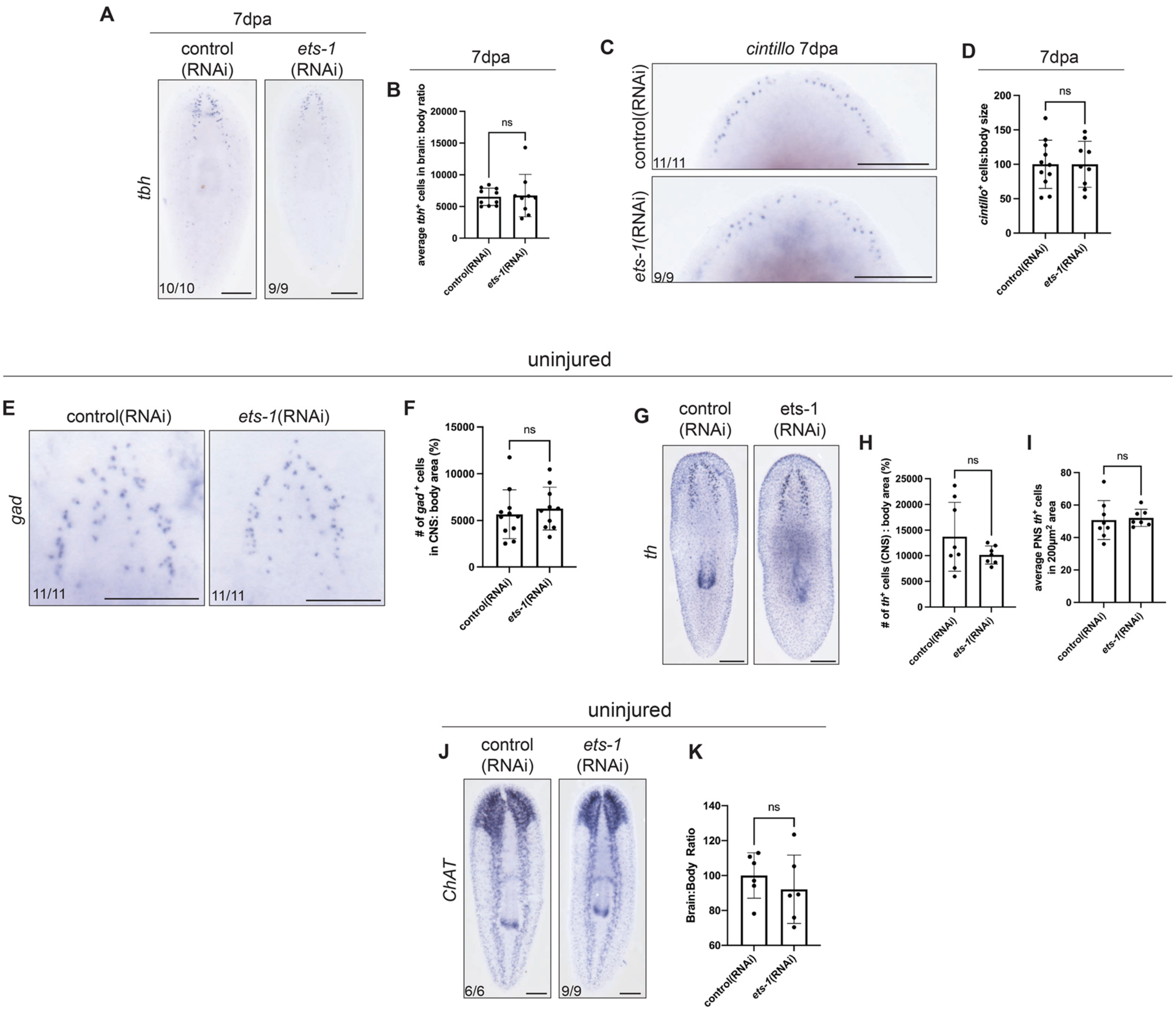

Fig. S7. *ets-1* knockdown does not affect neuronal cell number. (A) 7 dpa control and *ets-1*(RNAi) animals were subjected to ISH with *tyramine beta-hydroxylase* (*tbh*) riboprobe. (B) Quantification of *tbh*$^+$ cells in brain (compared to body size). (C) Regenerated control and *ets-1*(RNAi) animals subjected to ISH with sensory neuron marker *cintillo*. (D) Quantification of *cintillo*$^+$ cells compared to body size in control and *ets-1*(RNAi) animals. (E) Uninjured control and *ets-1*(RNAi) were subjected to ISH with *gad* riboprobe. (F) Quantification of *gad*$^+$ cell numbers in the CNS compared to body size in uninjured control and *ets-1*(RNAi) animals. (G) ISH of uninjured control and *ets-1*(RNAi) animals against riboprobe marker *th*. (H-I) Quantification of *th*$^+$ cells in CNS and PNS normalized to body size or specific area, respectively, in *ets-1*(RNAi) animals compared to control. (J) ISH of uninjured control and ets-1(RNAi) animals with riboprobe against *ChAT*. (K) Quantification of brain-to-body ratio (normalized to control). Unpaired t-test with Welch's correction. ns=not significant. Scale bar: 200 µm.

Development • Supplementary information

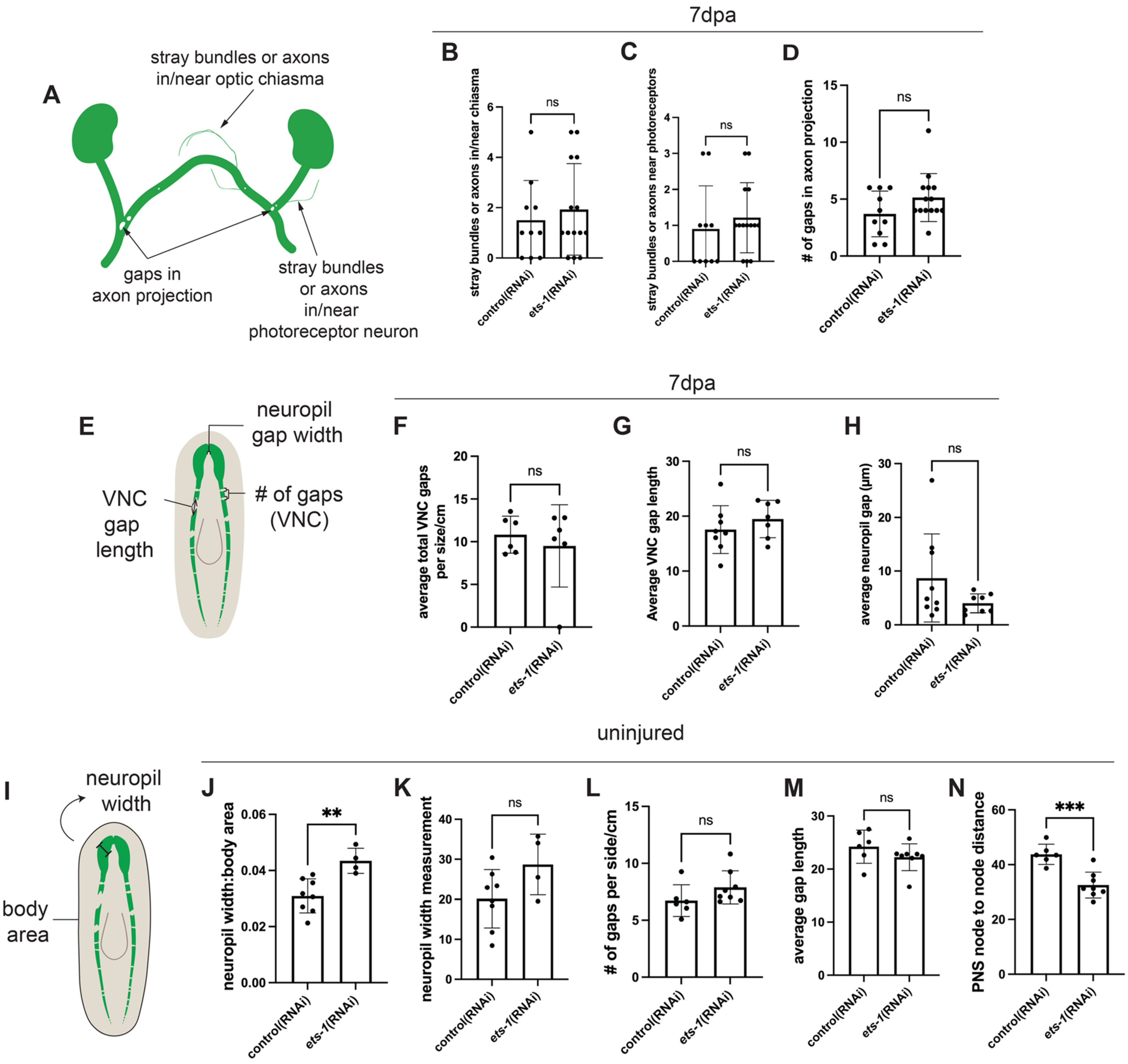

Fig. S8. *ets-1* knockdown affects neural architecture.

(A) Graphical illustration of criteria quantified individually in anti-Arrestin immunofluorescence images: stray bundles or axons in/near optic chiasm; stray bundles or axons in/near photoreceptor neuron; and gaps in axon projection. (B-D) Quantification of stray bundles or axons in or near the optic chiasm, near photoreceptors (left and right), and gaps in axon projections in control and *ets-1*(RNAi) animals. Each point is an individual animal. Aggregated data are in Fig. 6B. (E) Graphical illustration of criteria that were quantified from anti-Synapsin immunofluorescence images: gap length, number of gaps, and neuropil gap length. (F-H) Quantification of total gaps in VNC per cm, average gap size in VNC (µm), and average gap length between neuropils (µm) for regenerated control and *ets-1*(RNAi) animals. (I) Graphical illustration of additional criterion (beyond those shown in Fig. S8E) that was quantified from anti-Synapsin immunofluorescence images in uninjured animals: neuropil width (compared to body area). (J-N) Quantification of neuropil width compared to body area, average neuropil width measurement, number of total gaps in VNC per cm, average gap size in VNC (µm), and average PNS node-to-node distance for uninjured control and *ets-1*(RNAi) animals. Unpaired t-test with Welch's correction. **p-value≤0.01, *** p-value≤0.001, ns = not significant.

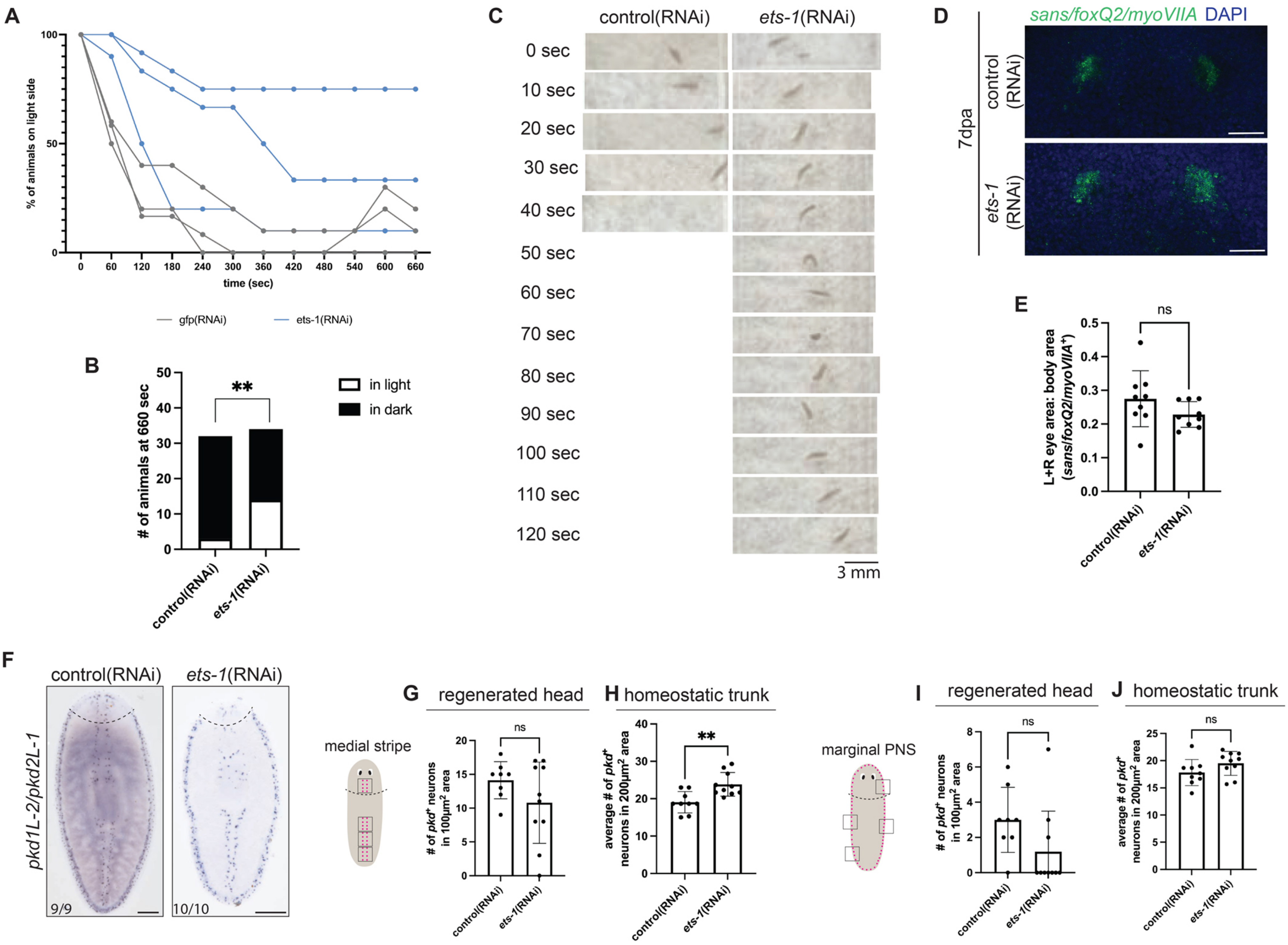

Fig. S9. Individual replicates of *ets-1*(RNAi) animals exhibited changes in phototaxis behavior.

(A) Graph showing percentage of animals from individual replicates of control and *ets- 1*(RNAi) animals that remained on the light side during filming across 660 seconds. Three replicates were completed (n=10-12 per replicate; aggregated data are in Fig.7B). (B) Quantification of the number of control and *ets-1*(RNAi) animals what were in the light or dark side of the dish at 660 seconds. Fisher's exact test. (C) Image stills of control and *ets-1*(RNAi) animals from Supplemental Video 1A-B. (D) Control and *ets- 1*(RNAi) regenerated animals were subjected to FISH with pooled photoreceptor neuron markers *sans*/*foxQ2*/*myoVIIA* (green) and DAPI (blue). (E) Quantification of photoreceptor neuron marker area (normalized to body size) in *ets-1*(RNAi) animals compared to control. (F) Control and *ets-1*(RNAi) regenerated animals were subjected to ISH with pooled sensory neuron marker *pkd1L-2/2L-1*. (G-H) Quantification of *pkd1L-2/2L-1*$^{+}$ cells in the medial stripe in the regenerated head and homeostatic trunk. (I-J) Quantification of *pkd1L-2/2L-1*$^{+}$ cells in the marginal PNS in regenerated heads and homeostatic trunks. Unpaired t-test with Welch's correction. **p-value≤0.01, ns = not significant. Scale bar (B) 50 µm, (D) 200 µm.

Development • Supplementary information

Table S1. List of cloning primers.

Click here to download Table S1

Table S2, List of protein sequences for phylogeny.

Click here to download Table S2

Table 3. List of RT-qPCR primers.

Click here to download Table S3

Table S4. Metadata of qPCR and RNA-seq of *ets-1*(RNAi) across multiple gene expression across in *cathepsin*⁺ cells (Dubey *et al., 20 22;* Fincher *et al.*, 2018; Plass *et al.*, 2018)**.**

Click here to download Table S4

Development • Supplementary information

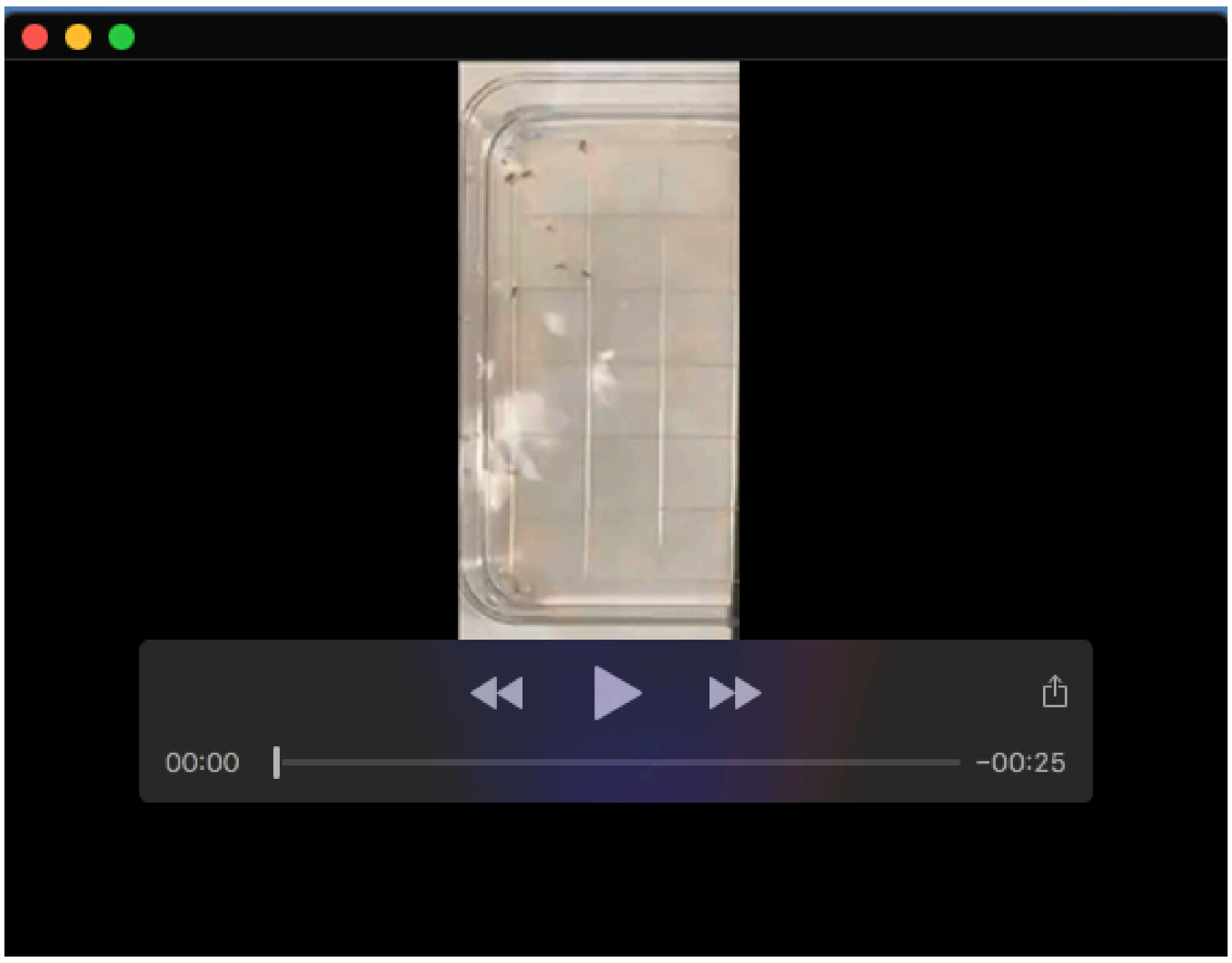

Movie 1A. *ets-1*(RNAi) animals have changes in negative phototaxis behavior. Control animals before head amputation in light/dark assay, 20x playback speed.

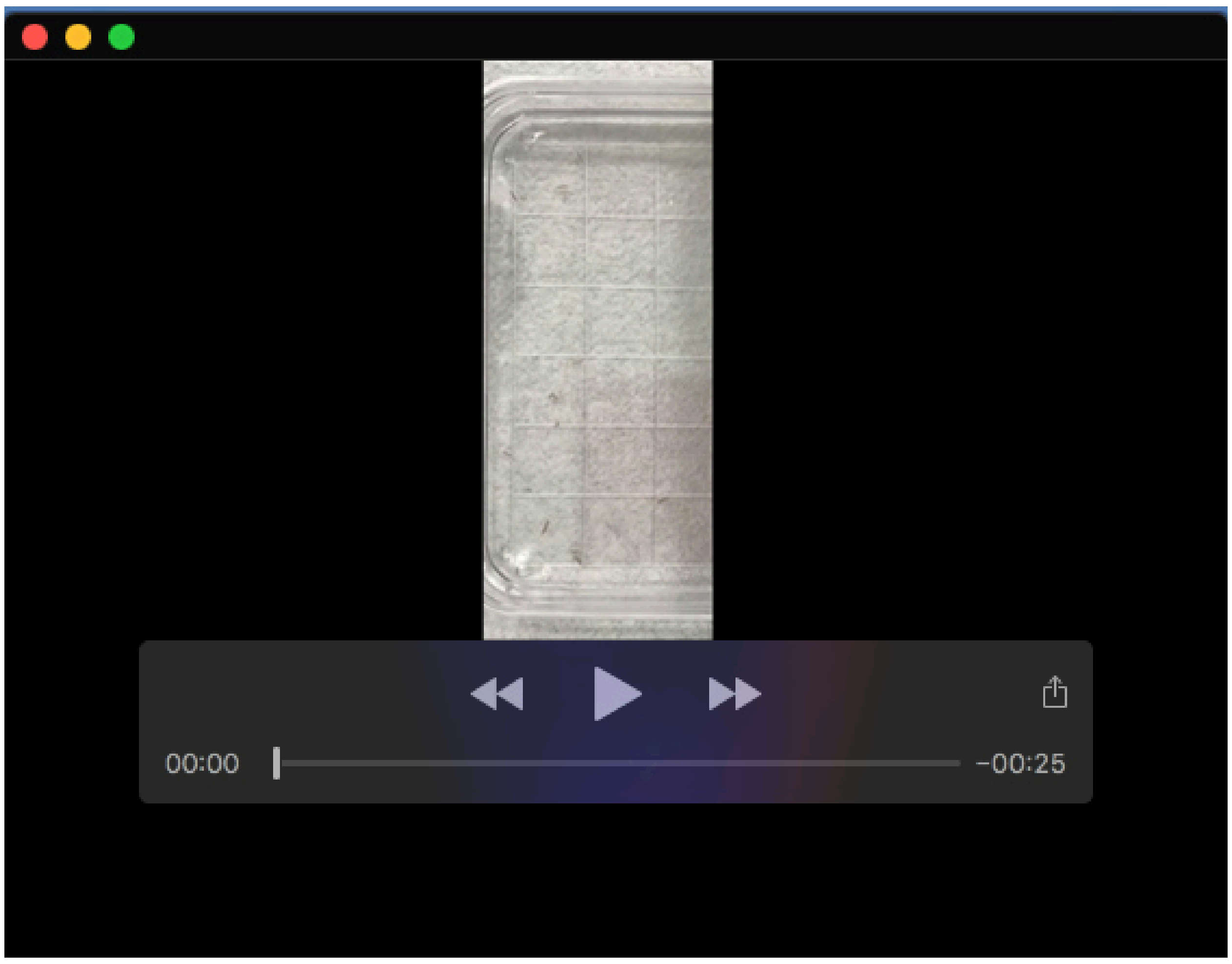

Movie 1B. *ets-1*(RNAi) animals have changes in negative phototaxis behavior. *ets-1*(RNAi) animals before head amputation in light/dark assay, 20x playback speed. Video only shows the light side of the dishes.

Development • Supplementary information

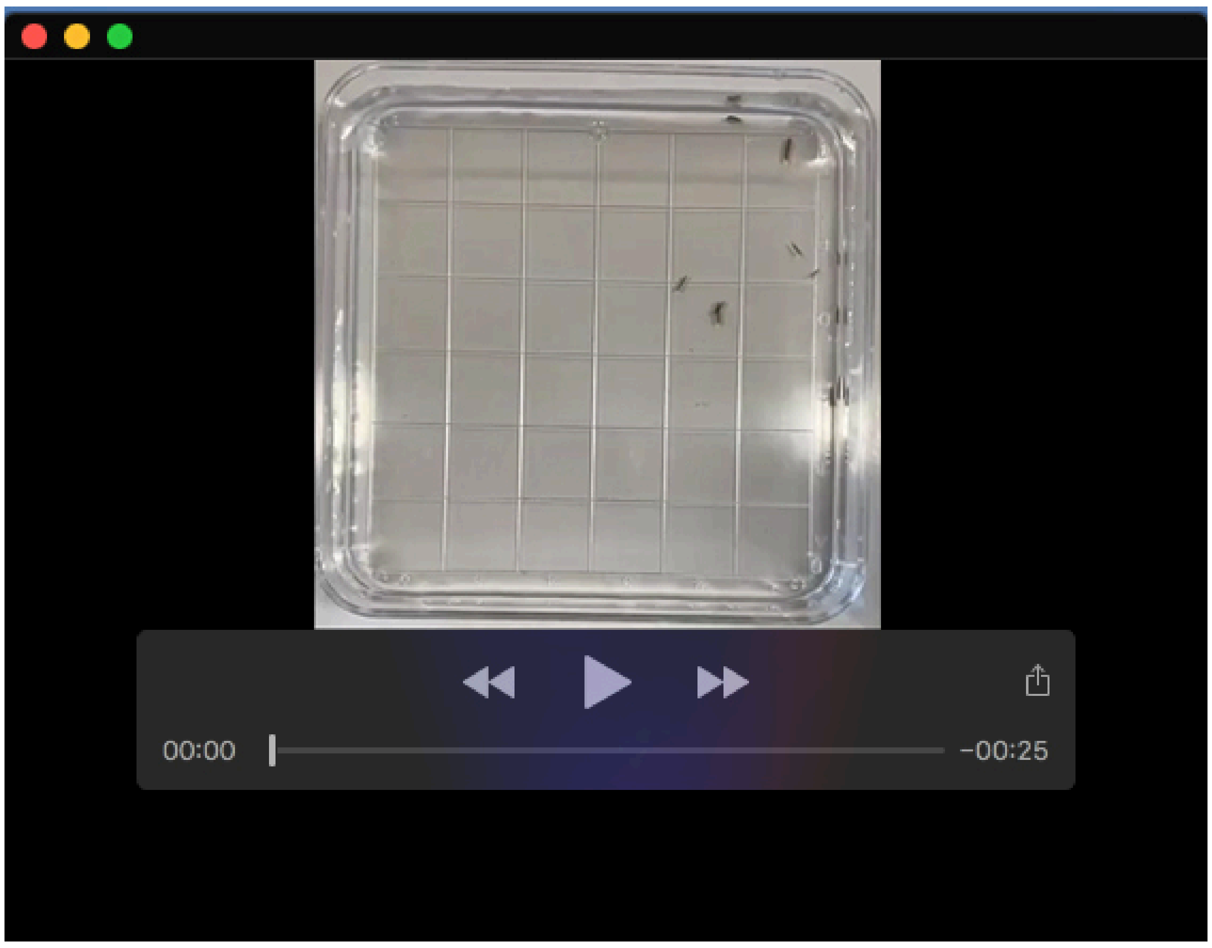

Movie 2A. ets-1(RNAi) animals exhibit locomotion defects.

Control animals before head amputation with no stimulus, 20x playback speed.

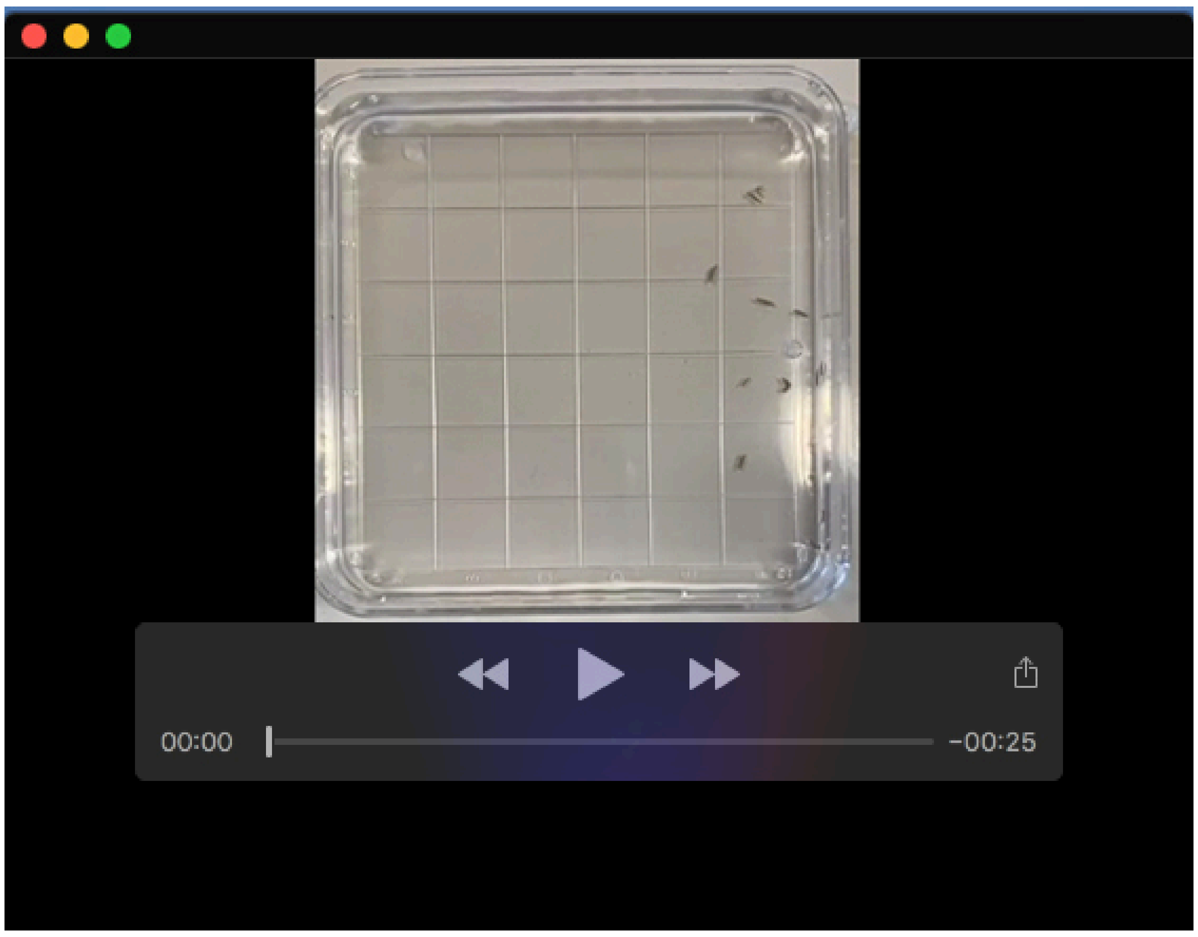

Movie 2B. ets-1(RNAi) animals exhibit locomotion defects.

ets-1(RNAi) animals before head amputation with no stimulus, 20x playback speed.

Development • Supplementary information
