# Supplementary material for: Ets-1 transcription factor regulates glial cell regeneration and function in planarians
Source: Development. 2023 Sep 14;150(18):dev201666. doi: 10.1242/dev.201666 (PMC10508700; doi:10.1242/dev.201666)
Supplement: Supplementary file 1 [file develop-150-201666_review_history.pdf]

Ets-1 transcription factor regulates glial cell regeneration and function in planarians

Bidushi Chandra, Matthew G. Voas, Erin L. Davies and Rachel Roberts-Galbraith
DOI: 10.1242/dev.201666

Editor: Kenneth Poss

Review timeline

Original submission:	1 February 2023
Editorial decision:	9 March 2023
First revision received:	17 July 2023
Accepted:	15 August 2023

Original submission

First decision letter

MS ID#: DEVELOP/2023/201666

MS TITLE: Ets-1 transcription factor regulates glial cell regeneration and function in planarians

AUTHORS: Bidushi Chandra, Matthew G Voas, Erin L Davies, and Rachel Roberts-Galbraith

I have now received all the referees' reports on the above manuscript, and have reached a decision. The referees' comments are appended below, or you can access them online: please go to BenchPress and click on the 'Manuscripts with Decisions' queue in the Author Area.

As you will see, the referees express interest in your work, indicating that it is important to understand glial regeneration in this key model system, and to explore the impact of this fascinating cell population on neural and whole-animal regeneration. They also have some significant criticisms and recommend a substantial revision of your manuscript before we can consider publication. All reviewers have constructive comments that I feel are reasonable. Of note, you will see that Reviewer #3 has strong concerns about the mechanistic depth of the study and the extent to which conclusions can be made about manipulations of ets-1 (and ova and soxb1-2) affecting specific cell populations, among other concerns. I realize that there are quite a number of reviewer comments to address, and that many of these would involve new experiments. However, if you are able to revise the manuscript to address the comments of all 3 referees, we will be happy receive a revised version of the manuscript to be re-reviewed by the original referees. Acceptance of your manuscript will depend on your addressing satisfactorily the reviewers' major concerns.

Please also note that Development will normally permit only one round of major revision. If it would be helpful, you are welcome to contact us to discuss your revision in greater detail. Please send us a point-by-point response indicating your plans for addressing the refereeâ€™s comments, and we will look over this and provide further guidance.

Please attend to all of the reviewers' comments and ensure that you clearly highlight all changes made in the revised manuscript. Please avoid using 'Tracked changes' in Word files as these are lost in PDF conversion. I should be grateful if you would also provide a point-by-point response detailing how you have dealt with the points raised by the reviewers in the 'Response to Reviewers' box. If you do not agree with any of their criticisms or suggestions please explain clearly why this is so.

© 2023. Published by The Company of Biologists under the terms of the Creative Commons Attribution License (https://creativecommons.org/licenses/by/4.0/).

Reviewer 1

Advance summary and potential significance to field

In this manuscript, the authors used planarians as model organisms to study the role of glial cells during development and regeneration. Through ISH, the authors demonstrated that gliogenesis occurs after neurogenesis during development and head regeneration. Disruption of neuronal regeneration by ovo and soxb1-2 RNAi impeded the expression of glial markers. In addition, the authors optimized the ets-1 RNAi paradigm to impair glial cells in planarian and found knocking down ets-1 affected the expression of several neuronal markers and the organization of neurons. In the end, the authors provide interesting locomotion and phototactic defect in ets-1 RNAi planarians, suggesting potential defects of neuronal functions in these animals.

This is a very descriptive paper, with most phenotypic analysis at the gene expression level. The interrelationship between neurons and glial cells suggested by this manuscript does not add much novel knowledge to the regeneration or developmental field. In addition, the authors did not suggest novel mechanisms to explain how glial and neuron shape each other during development or regeneration. The methodology applied by the authors cannot fully support the conclusion they made in the manuscript. All of these concerns dampen the enthusiasm for publishing this paper in Development.

Comments for the author

Review:

In this manuscript, the authors used planarians as model organisms to study the role of glial cells during development and regeneration. Through ISH, the authors demonstrated that gliogenesis occurs after neurogenesis during development and head regeneration. Disruption of neuronal regeneration by *ovo* and *soxb1-2* RNAi impeded the expression of glial markers. In addition, the authors optimized the ets-1 RNAi paradigm to impair glial cells in planarian and found knocking down *ets-1* affected the expression of several neuronal markers and the organization of neurons. In the end, the authors provide interesting locomotion and phototactic defect in *ets-1* RNAi planarians, suggesting potential defects of neuronal functions in these animals.

This is a very descriptive paper, with most phenotypic analysis at the gene expression level. The interrelationship between neurons and glial cells suggested by this manuscript does not add much novel knowledge to the regeneration or developmental field. In addition, the authors did not suggest novel mechanisms to explain how glial and neuron shape each other during development or regeneration. The methodology applied by the authors cannot fully support the conclusion they made in the manuscript. All of these concerns dampen the enthusiasm for publishing this paper in *Development*.

General comments:

1. The authors did not include the uninjured group for most experiments they showed in this manuscript. I found it difficult to interpret some of the results in the manuscript without these controls. Some statements made by the authors are not well supported by the data they provided (for example, the last sentences of page 6).

2. The authors use RNAi to assess neuron-dependent glia regeneration and glia-dependent neuron organization and activity and make the key conclusions based on RNAi results for the manuscript. I have 2 main concerns with the RNAi experiments here. 1). The authors use *ovo* RNAi and *soxb1-2* RNAi to perturb the regeneration of neurons and assess the expression of *Estrella*. How specific are these perturbations to neurons in these RNAi-fed animals? Can the authors cite or show data to demonstrate that RNAi feeding does not affect other regenerating cell types, including directly affecting glial cells themselves? Or the authors need to provide data to clearly demonstrate that disruption of other tissues by RNAi does not affect glial regeneration in their model. The use of *Ets-1* RNAi to assess glia-dependent neuron activity raised similar concerns. 2). The authors did not

© 2023. Published by The Company of Biologists under the terms of the Creative Commons Attribution License (https://creativecommons.org/licenses/by/4.0/).

include uninjured/intact animals feeding with RNAi as a control in most of their RNAi experiments. It is extremely important here to demonstrate that perturbing gene expression itself does not affect the maintenance of neurons or glia in intact planarians.
3. Some images chosen by the authors in the manuscript do not support their quantification results (Like fig. 3C, fig. 5A, fig. 6A)

Specific comments:

1. In fig 1A, the authors use *ChAT* as a marker to show neuron establishment in the regenerating head. It is better to use a pan-neuron marker instead.
2. The author showed in fig. 1C that the dynamism of if-1 and calamari are very different. What is the rationale behind using pooled *if-1* and *calamari* probe for ISH shown in fig.1A?
3. At 15 dpa, pooled *if-1* and *calamari* probes gave a weaker signal than 11 dpa. Can the author explain this phenomenon?
4. It is difficult to tell the morphology of *estrella*$^{+}$ cells from the ISH images shown in fig 1B. The authors need to provide better images, use other experimental approaches, or describe how they access the morphology.
5. Does the "time zero" in fig 1C mean "uninjured" or "zero hours after amputation"? Can the authors comment on why the expression of glial markers is transiently downregulated immediately after injury? Can the authors provide ISH images at early injury time points to show this transient downregulation?
6. The authors mentioned on page 9, paragraph 1, that "*estrella*+ glial cells are present in the vicinity of the photoreceptor neurons of the eyespots and the ciliated sensory neurons along the dorsal midline and lateral margins of the body." Again, the authors need to state clearly whether this sentence is describing the association of glial cells with neurons in uninjured animals or describing the spatial distribution of new-borne glia. In addition, the authors need to better define "vicinity" or quantitively show "vicinity" since the distance between neurons and *estrella*+ glial cells in fig 3A&C are very different.
7. Can the authors clearly state in the main text or figure legend which cells are counted as "*estrella*+ cells in proximity to *pkd*+ *cells*" in fig 3C? In addition, there are clearly *estrella*+ cells that are not in proximity to *pdk*+ cells. Does knocking down *soxb1-2* affect those glial cells?
8. Can the authors better explain how they optimized the *ets-1* feeding paradigm and how they decided which feeding paradigm is optimum for their experiment (except the fact that they see minimal *estrella* expression with their current feeding paradigm)?
9. In the homeostasis paradigm (fig.4B), authors collect uninjured samples at 19 days (7 days post last feeding) but in the regeneration paradigm (fig.4C), the authors collect samples at 26 days (14- day post last feeding). Can the authors please comment on why they use such paradigms and assess *ets-1* levels at 7 and 14 days post-last feeding?
10. According to Fig.4J, the *calamari* expression level is partially recovered in the regenerating head and pre-existing VNC after amputation. Is this observation consistent across all animals? Can authors comment on this phenomenon? Does injury induce *calamari* expression?
11. Is the RT-PCR in fig. 4K & fig. 6C performed on uninjured or regenerating animals? Whole animal or only head region? Please also provide ISH data for *if-1, npp3,* and *spp12*.

12. The FISH result in Fig. 5A is not consistent with the ISH result in Fig.4. FISH of *calamari* showed minimal detectable signals (close to zero cells expressing *calamari*). However, the ISH of *calamari* in Fig. 4J suggested the existence of several *calamari*+ cells in the regenerating head. The image in Fig.5A does not well reflect the quantification in fig. 5B-5F. Please also use arrows to indicate cells belonging to each counting category.
13. With the current available single-cell dataset, can authors better comment on/suggest the identity of the different glial populations shown in fig.5B-F?
14. Are 12% and 28% gene expression changes shown in fig.S6B sufficient to lead to pigmentation changes in planarians?
15. Please provide FISH images of *gad* in fig. 6.
16. Please use the arrow to indicate cells belonging to each counting category for fig. 6H, fig. S7A & B.

17. The authors mentioned on page 16, that "We did not see phenotypes that would indicate widespread neuronal cell death or failure to specify neurons during regeneration after reduction in

© 2023. Published by The Company of Biologists under the terms of the Creative Commons Attribution License (https://creativecommons.org/licenses/by/4.0/).

glial number.". Please provide data to support this statement.
18. In figure 7, the authors showed interesting locomotion defects of planarians fed with *ets-1* RNAi.

Beyond the impairment of neuronal function suggested by the authors in the manuscript, it is also very likely due to muscle defects in animals. Can the author exclude this possibility by evaluating the effect of *ets-1* RNAi on muscles?

Reviewer 2

Advance summary and potential significance to field

In this well-written paper, Chandra and colleagues address the development, regeneration, and function of glia in the planarian S. mediterranea. In humans and more widely studied models, glia play important roles in nervous system development and function, but also have inhibitory roles in regeneration by promoting formation of a Â"glial scar.Â" Understanding glial biology in systems like planarians that do not form scars is therefore potentially important for regenerative medicine. Prior to this work, multiple markers for planarian glia had been identified, and superficial efforts had identified ets-1 as a potential regulator of glia differentiation, but the functional consequences of glia loss had not been investigated. First, Chandra et al. demonstrated that glia differentiation occurred after neuron development, by analyzing published RNA-Seq data, and by conducting an elegant and thorough series of in situ hybridization experiments on both regenerating planarians and planarian embryos and hatchlings.
They then showed that inhibiting differentiation of eye neurons during homeostasis and regeneration also delayed glia development, and that inhibiting midline and marginal sensory neuron differentiation caused a modest (but statistically insignificant) decrease in glia. Next, they convincingly showed (using multiple markers) that knocking down ets-1 inhibited glia differentiation, and that this affected estrella+ and calamari+ glia subpopulations differently. They went on to show that ets-1 RNAi disrupted neural gene expression and organization, although no specific neuron type seemed to be reduced in number, and many trends were not statistically significant. Finally, ets-1 RNAi also caused behavioral defects, including increased inchworming movement and decreased negative phototaxis.

The authors conclude that the sequence of neurogenesis followed by gliogenesis is conserved in planarians, that (sensory) neurons might be required for gliogenesis, that ets-1 plays a conserved role in gliogenesis, and that glia subtly influence neural gene expression, CNS organization, and neural function.
With the caveat that some of the effects of ets-1 inhibition might be due to dysregulation of other phagocytic cell types (poorly understood cathepsin+ cells), the study is the first to identify functional role(s) for planarian glia, building on the PIÂ's and othersÂ' previous work. This sets the stage for multiple future directions, which the authors outline in their discussion. The work is a novel and substantial advancement in understanding of glia in an emerging regeneration model. However, the authorsÂ'
conclusions are somewhat tentative, based on data with small sample sizes and/or statistically insignificant trends. Also, the relationship between the behavior phenotypes observed and the effects on neuronal subpopulations is unclear. The conclusions could be strengthened by addressing these issues in several ways.

Comments for the author

Major recommendations

1. Provide more insight into whether inhibiting glia birth/function affects neuron numbers.

In Figure 6A, ets-1 inhibition reduces brain size by about 20%. However, expression of gad, th, and tph (neuron subtype markers) increased by qPCR, gad+ cell numbers did not change but their locations were abnormally patterned, and th+ cell numbers increased significantly in PNS, and insignificantly in CNS/pharynx. This leads to the tentative conclusion (p. 16) that "inhibition of ets-

© 2023. Published by The Company of Biologists under the terms of the Creative Commons Attribution License (https://creativecommons.org/licenses/by/4.0/).

1, *possibly* due to loss of glial cells, *broadly but subtly* impacts planarian neuronal gene expression and CNS organization."
While this is true, the data do not explain why the brain might be smaller, or why ets-1 inhibition yields behavioral phenotypes. The authors should consider:

- Assessing the reciprocal effect of ets-1 knockdown on photoreceptor cell numbers (ovo/sans/foxQ2/myoVIIA+ cell numbers). Are smaller eyes/fewer photoreceptors responsible for reduced light responsiveness?
- Assessing the reciprocal effects of ets-1 knockdown on sensory neuron cell number (soxB1-2+, pkd2L-1+, pkd1L-2+ cell numbers). Are fewer sensory neurons responsible for reduced light responsiveness or motility?
- Assessing serotonergic neuron numbers (pitx+, lhx1/5-1+, etc.) since genetic ablation of these neurons also can cause inchworming phenotypes.
- Assessing markers for other neuronal subtypes.

While doing all of these experiments is probably beyond the scope of the manuscript, determining whether ets-1 reduces CNS/PNS neuron numbers, especially those that might be linked to the observed behavior phenotypes, would strengthen the manuscript considerably.

2. Provide more insight into whether inhibiting neurogenesis affects gliogenesis.

On page 9, section 2 of Results, the authors suggest that "Glial regeneration in sensory structures *might* depend on neurons." It seems that this tentative conclusion is based on the fact that although there was a significant reduction in eye glia, there was only a "consistent but nonsignificant decrease in regeneration of PNS glia." While it seems possible there is a decrease during homeostasis, a downward trend is questionable in the regeneration data (Fig. 3C). This could be addressed in one or more of the following ways, although there may be other methods:

- Quantify more animals/regions or larger areas to reduce variability.
- Assess glia numbers later, when estrella+ cells should be more abundant (Fig. 1).
- Use calamari or another marker to quantify glia, since it seems that calamari+ cells are about twice as abundant (Fig. 5B-C).
- Analyze brain regions in soxB1-2 animals, since soxB1-2 reduces brain size (Ross et al., 2018), and the authors were able to quantify brain glia in Fig. 5. These samples might already be available.
- Extend analysis to CNS (brain/VNC) neurons (RNAi of nkx2.1, tcf-1, etc.).
- Knock down nou darake or tec-1 to increase neuron numbers. Would this be accompanied by more glia?

Minor recommendations

1. ets-1 knockdown seemed to have modest but insignificant effects on photoreceptor axons/connectivity (Fig. S7G-J). If the total number of defects per animal is added together, does a phenotype become more obvious/significant?

2. The authors stated (p. 16) that "we did not see phenotypes that would indicate widespread neuronal cell death…" without specifying what phenotypes would indicate this, since they only assessed a limited number of neuronal markers and did not assess cell death directly. Could the authors clarify what phenotypes would indicate widespread death?

3. The abstract states that "…ets-1(RNAi) impairs nervous system architecture…" but the title of Figure S7 is "ets-1 knockdown does not affect neural architecture." These statements conflict.

4. It is not clear how many days elapsed after feeding and amputation before the authors assessed the dependence of glial cell regeneration on neurons (Figure 3). The authors could consider adding an RNAi timeline as in Figure 4C to Figure 3 or clarify in the figure legend.

5. Obvious examples of inchworming are not apparent in the videos. Can the authors provide a higher magnification example?

© 2023. Published by The Company of Biologists under the terms of the Creative Commons Attribution License (https://creativecommons.org/licenses/by/4.0/).

Reviewer 3

Advance summary and potential significance to field

This study by Chandra et al explores the role of planarian glial during regeneration and the function of the transcription factor Ets-1 during glial cell regeneration in the planarian Schmidtea mediterranea. The authors show glial cell development and regeneration occur after the generation and regeneration of neurons and suggest that neurons are required for the emergence of glial cells. They show that Ets-1 is required for the maintenance of existing glial cells in uninjured tissues and for the regeneration of new glia after injury. They conclude that ets-1 RNAi non-cell-autonomously impacts neuronal gene expression neuropil size, and animal movement.

This is a well-executed and significant study that explores the role of glial cells and the functional relationship between glia and neurons during development and regeneration. This reviewer has only few suggestions focused on data analysis and presentation that will improve the strengths of the study.

Comments for the author

1- The authors used ovoRNAi to disrupt neurogenesis and to show that neurons are required for glial development and regeneration. The evidence for this conclusion is insufficient and inconclusive. Quantifications in figures 3B and S2A are less convincing in the absence of error bars and N numbers. Normalized quantifications and data presentation similar to the quantifications in 3C are needed to support the conclusion. In addition, it is unclear why nearly 1/3 of the scored eyes had no estrella expression in the control RNAi condition although 100% of the control eyes were positive for sans. How much of these quantifications depend on a user defined threshold for Estrella or sans expression level? Quantifying normalized fluorescence signal in addition to binary scoring of cells as positive or negative would address this concern. If confirmed, how do the authors interpret the lack of estrella expression in a subset of the control RNAi sample.

2- The effects of ets-1 on glial cells is remarkable, but opens a question about the differential roles of calamari and estrella in different glial cell populations. The data suggest that calamari and/or calamari expressing cells are the main targets of ets-1. What is known about calamari versus estrella and the cells they express? In light of the data presented in figure 5, it would be informative to check the expression of calamari after ovoRNAi. Could calamari expressing cells be more sensitive to the generation and regeneration of neurons?

3- Figures 1 and 2 include nice ISH showing that development and regeneration of neurons precede glia. Including error bars and statistics in these quantifications would help strengthen this conclusion.

4- The effects of glial cells on neuron gene expression is intriguing and I agree with the authors that additional future studies are needed to better understand the gene expression changes observed in neurons. However, and although ets-1 has been previously studied in glial cells, the study did not establish that manipulating ets-1 results in “non-autonomous” effects on neurons. Detailed expression analysis for ets-1 in neurons and glia beyond what is shown in figure 4A will help address this question.

5- The effects of glial cells on animal behavior is also of interest. The methods section states that experiments were performed for 3 biological replicates. But it is difficult to appreciate how variable of consistent behaviors are in 7A in the absence of error bars or statistics. It is also important to normalize the data in 7B to show % of animals per replicate with error bars instead of or in addition to number of animals.

6- The study clearly shows a role for ets-1 during glial cell maintenance in figure 4 but those findings are not quantified.

© 2023. Published by The Company of Biologists under the terms of the Creative Commons Attribution License (https://creativecommons.org/licenses/by/4.0/).

7- The RNAi Methods section states “The number of animals alive was quantified as detailed in Fig S3A”. I believe this refers to figure S5.

First revision

Author response to reviewers' comments

We thank all three reviewers for the time and effort spent thoughtfully reviewing this manuscript. The suggestions and critiques of all reviewers helped to make this manuscript better. We completed many new experiments to strengthen and clarify our main conclusions. We also made changes in the writing and presentation of our work to more clearly explain our work. Responses to individual reviewer comments follow.

Reviewer 1 comments for the authors:
In this manuscript, the authors used planarians as model organisms to study the role of glial cells during development and regeneration. Through ISH, the authors demonstrated that gliogenesis occurs after neurogenesis during development and head regeneration. Disruption of neuronal regeneration by ovo and soxb1-2 RNAi impeded the expression of glial markers. In addition, the authors optimized the ets-1 RNAi paradigm to impair glial cells in planarian and found knocking down ets-1 affected the expression of several neuronal markers and the organization of neurons. In the end, the authors provide interesting locomotion and phototactic defect in ets-1 RNAi planarians, suggesting potential defects of neuronal functions in these animals.

This is a very descriptive paper, with most phenotypic analysis at the gene expression level. The interrelationship between neurons and glial cells suggested by this manuscript does not add much novel knowledge to the regeneration or developmental field. In addition, the authors did not suggest novel mechanisms to explain how glial and neuron shape each other during development or regeneration. The methodology applied by the authors cannot fully support the conclusion they made in the manuscript. All of these concerns dampen the enthusiasm for publishing this paper in *Development*.

We appreciate the reviewer’s comments and assessment of the paper. We argue that many of the descriptive elements of the manuscript are necessary, as glia have not been studied in any depth in planarians to this point. We do show that *ets-1* is required for maintenance and regeneration of glia. This is the first gene comprehensively shown (with multiple markers and rigorous quantification) to affect glial gene expression and numbers. We also show several impacts of *ets-1(RNAi)* on behavior and neuronal biology, giving the first look at potential roles for planarian glia in neuronal physiology. We also show—with several added experiments—that neurons are required for glial arrival into the nervous system. We agree that glial and neuronal influence on one another will certainly be productive next steps, but we believe that the current manuscript marks an important, consequential, and complete step forward. We believe that our discoveries are likely to accelerate the study of glia in planarians and we wish to share our work with the research community at this time so that others can join us in studying these fascinating cells.

We appreciate the many constructive suggestions and critiques of the reviewer and have worked to address as many of them as we possibly could. Changes based on these excellent suggestions are detailed below.

General comments:
1. The authors did not include the uninjured group for most experiments they showed in this manuscript. I found it difficult to interpret some of the results in the manuscript without these controls. Some statements made by the authors are not well supported by the data they provided (for example, the last sentences of page 6).

We completed experiments with uninjured animals. The new data include:

© 2023. Published by The Company of Biologists under the terms of the Creative Commons Attribution License (https://creativecommons.org/licenses/by/4.0/).

- Supplemental Figure 1B: Images provided of ISH with neural and glial markers on uninjured animals compared to 36 hpa animals.
- Supplemental Figure 4F-L: Images provided of an uninjured *ets-1*(RNAi) paradigm, ISH of *ets-1*(RNAi) with *calamari* and *estrella* riboprobes, and respective quantifications.
- Supplemental Figure 7E-K: Images provided of ISH performed on uninjured *ets-1*(RNAi) animals with multiple neuronal markers (*chat, gad, th*) and respective quantifications.
- Figure 6G-H; Supplemental Figure 8: Images provided of anti-Synapsin immunofluorescence on uninjured ets-1(RNAi) animals and respective quantifications

We do wish to emphasize that uninjured planarians still use stem cells to add new cells during animal growth and to homeostatically replace cells at some basal rate. Thus, even in an uninjured animal, gliogenesis and neurogenesis are still occurring; often genes that impact such a process will be evident in both regeneration and non-injury paradigms.

2. The authors use RNAi to assess neuron-dependent glia regeneration and glia-dependent neuron organization and activity and make the key conclusions based on RNAi results for the manuscript. I have 2 main concerns with the RNAi experiments here. 1). The authors use ovo RNAi and soxb1-2 RNAi to perturb the regeneration of neurons and assess the expression of Estrella. How specific are these perturbations to neurons in these RNAi-fed animals? Can the authors cite or show data to demonstrate that RNAi feeding does not affect other regenerating cell types, including directly affecting glial cells themselves? Or the authors need to provide data to clearly demonstrate that disruption of other tissues by RNAi does not affect glial regeneration in their model. The use of Ets-1 RNAi to assess glia-dependent neuron activity raised similar concerns. 2). The authors did not include uninjured/intact animals feeding with RNAi as a control in most of their RNAi experiments. It is extremely important here to demonstrate that perturbing gene expression itself does not affect the maintenance of neurons or glia in intact planarians.

ovo is specifically expressed in eyespot cells (photoreceptors and pigment cup cells) and eyespot progenitors (Lapan and Reddien, *Cell Reports* 2012). There is also no expression of *ovo* in glia in published single-cell sequencing data (Fincher, et al *Science* 2018; Plass, et al *Science* 2018). The published data, together, support that *ovo(RNAi)* is quite specific to pigment cup cells and neurons of the eye.

soxb1-2 is expressed in neurons, epidermal cells, and neural/epidermal progenitors according to published work (Ross, et al *Developmental Cell* 2018) and single cell sequencing data (Fincher, et al *Science* 2018; Plass, et al *Science* 2018). There is no evidence of *soxb1-2* expression in glia in any of those datasets.

We added a note in the text to more clearly indicate the specificity of both genes' expression and known function.

As described above, we repeated many of our experiments in intact animals to show which genes have regeneration-specific defects and which phenotypes occur in both homeostasis and regeneration contexts. We note that planarians experience turnover of mature cell types at some basal rate and therefore animals in homeostasis would be expected to have neurogenesis and gliogenesis occurring at some level.

3. Some images chosen by the authors in the manuscript do not support their quantification results (Like fig. 3C, fig. 5A, fig. 6A)

For some of these experiments, we increased N. After revisiting all experiments with old and new data, we double checked that all images are representative of the range of phenotypes/patterns seen and consistent with conclusions drawn from the experiment.

Specific comments:
1. In fig 1A, the authors use ChAT as a marker to show neuron establishment in the regenerating head. It is better to use a pan-neuron marker instead.

We have yet to identify a truly pan-neuronal marker in planarians and most broad neural markers are expressed in many but not all neurons. Each synapsin and synaptotagmin gene is expressed in

© 2023. Published by The Company of Biologists under the terms of the Creative Commons Attribution License (https://creativecommons.org/licenses/by/4.0/).

some but not all neurons, for example. In our analysis, we therefore used three of the most widely expressed neural markers, *synt1*, *ChAT*, and *PC2* (two of these are in the supplement), and note that *PC2* and *ChAT* are expressed in non-overlapping cell types.

2. The author showed in fig. 1C that the dynamism of if-1 and calamari are very different. What is the rationale behind using pooled if-1 and calamari probe for ISH shown in fig.1A?

if-1 and *calamari* probes are not as strong as *estrella* and *if-1* and *calamari* have been pooled in other experiments that focus on glial presence/absence (e.g. Scimone, et al *Current Biology* 2016). We find that *if-1* and *cali* are more similar in expression dynamics than either of those markers and *estrella*. Therefore, we combined those probes in some experiments when we wanted a stronger signal to allow us to also visualize peripheral glia. We have also separated the probes in experiments where we wished to evaluate each gene's expression individually.

3. At 15 dpa, pooled if-1 and calamari probes gave a weaker signal than 11 dpa. Can the author explain this phenomenon?

The signal is weaker, but ISH is also semi-quantitative, so this could just be within the bounds of expected variability between samples. Looking across all the animals in this experiment, we cannot definitively say that the weaker signal is consistent or significant.

4. It is difficult to tell the morphology of estrella+ cells from the ISH images shown in fig 1B. The authors need to provide better images, use other experimental approaches, or describe how they access the morphology.

We previously showed the morphology of planarian glia in more detail with FISH and electron microscopy (Roberts-Galbraith, et al, *eLife* 2016). Glial morphology was also shown by immunofluorescence in (Wang, et al, *eLife* 2016).

Below are images showing morphology changes of *estrella*+ cell over different time points after amputation. We are currently pursuing quantitative methods to describe cell shape in glia and plan to pursue this trait of glial cells in our future work.

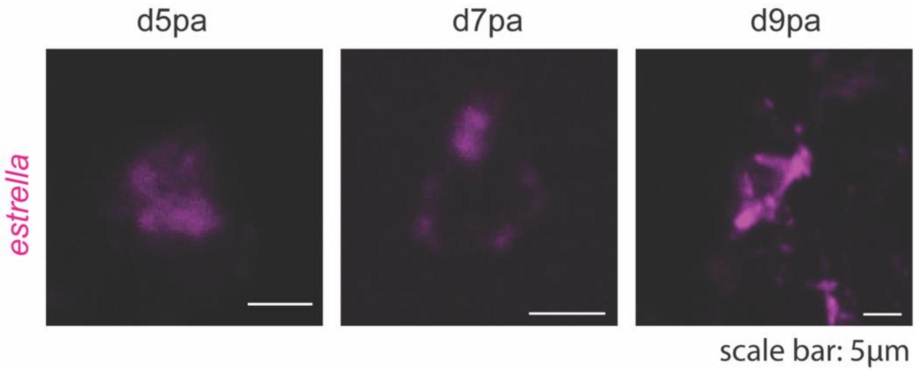

5. Does the "time zero" in fig 1C mean "uninjured" or "zero hours after amputation"? Can the authors comment on why the expression of glial markers is transiently downregulated immediately after injury? Can the authors provide ISH images at early injury time points to show this transient downregulation?

Time zero means zero hours after amputation. We do not yet know why glial markers are transiently downregulated immediately after injury but this trend was seen with several different glial-expressed genes in our prior transcriptomic analysis (Roberts-Galbraith, et al, *eLife* 2016). We speculate that this is a change of state analogous to—but functionally different from—the well-known state changes that occur in glia in non-regenerative organisms after injury. We also think that understanding these changes in gene expression will be a really promising future direction and plan to pursue this question in our future work. We also clarified the definition of time zero in the graph.

© 2023. Published by The Company of Biologists under the terms of the Creative Commons Attribution License (https://creativecommons.org/licenses/by/4.0/).

6. The authors mentioned on page 9, paragraph 1, that “estrella+ glial cells are present in the vicinity of the photoreceptor neurons of the eyespots and the ciliated sensory neurons along the dorsal midline and lateral margins of the body.” Again, the authors need to state clearly whether this sentence is describing the association of glial cells with neurons in uninjured animals or describing the spatial distribution of new-borne glia. In addition, the authors need to better define “vicinity” or quantitively show “vicinity” since the distance between neurons and estrella+ glial cells in fig 3A&C are very different.

We quantified only glial cells *adjacent to* the eyespot (considering the three-dimensional placement of the eye). We clarified this issue in the main text and methods section.

7. Can the authors clearly state in the main text or figure legend which cells are counted as “estrella+ cells in proximity to pkd+ cells” in fig 3C? In addition, there are clearly estrella+ cells that are not in proximity to pdk+ cells. Does knocking down soxb1-2 affect those glial cells?

For medial glia, we counted only cells present *in the medial stripe* or which had *clear contact with medial stripe pkd+ neurons*. For lateral glia, we counted cells that were *intermixed with* lateral *pkd*$^{+}$ cells or which *had contact with* lateral *pkd*$^{+}$ cells. We clarified this issue in the main text.

8. Can the authors better explain how they optimized the ets-1 feeding paradigm and how they decided which feeding paradigm is optimum for their experiment (except the fact that they see minimal estrella expression with their current feeding paradigm)?

We changed both dosage (µg/feeding) and number of feedings to optimize the paradigm. We looked for knockdown efficiency and also the variability of the knockdown between animals to determine the optimal paradigm. We found that a lower dosage or number of feedings could result in greater variability of the phenotypes that we see or even no phenotype at all, which may be why the effect of *ets-1(RNAi)* on glia was not observed in He, et al (*Development*, 2017).

9. In the homeostasis paradigm (fig.4B), authors collect uninjured samples at 19 days (7 days post last feeding) but in the regeneration paradigm (fig.4C), the authors collect samples at 26 days (14-day post last feeding). Can the authors please comment on why they use such paradigms and assess ets-1 levels at 7 and 14 days post-last feeding?

We typically wait 5-7 days after feeding to process animals for fixation or amputation, due to food that remains in the gut in the days immediately after feeding. After amputation, we wait the number of days indicated until brain or glial regeneration is sufficient for our experiments. We completed the RT-qPCR after amputation and saw a strong decrease in *ets-1* transcript levels, indicating that there is no “rebound” in mRNA despite the 14 days since the last feeding.

10. According to Fig.4J, the calamari expression level is partially recovered in the regenerating head and pre-existing VNC after amputation. Is this observation consistent across all animals? Can authors comment on this phenomenon? Does injury induce calamari expression?

We emphasize that *calamari* levels are still decreased in *ets-1(RNAi)* animals compared to controls after amputation and this is evident in ISH, FISH, and RT-qPCR experiments.

One possibility is that the homeostasis and post-amputation experiments were performed separately and thus ets-1(RNAi) occurred at different penetrance in the two experiments. The second possibility is that there could be some interaction of gene perturbation and injury that alters *calamari* expression, which we do not yet understand. We do think that injury-responsiveness of glial gene expression is important, but we plan to pursue this in future research.

11. Is the RT-PCR in fig. 4K & fig. 6C performed on uninjured or regenerating animals? Whole animal or only head region? Please also provide ISH data for if-1, npp3, and spp12.

We specified the injury status of animals for RT-qPCR in the text and figures. We are also including ISH data for *npp3* and *spp12* as requested (see Figures 5P-T).

© 2023. Published by The Company of Biologists under the terms of the Creative Commons Attribution License (https://creativecommons.org/licenses/by/4.0/).

12. The FISH result in Fig. 5A is not consistent with the ISH result in Fig.4. FISH of calamari showed minimal detectable signals (close to zero cells expressing calamari). However, the ISH of calamari in Fig. 4J suggested the existence of several calamari+ cells in the regenerating head. The image in Fig.5A does not well reflect the quantification in fig. 5B-5F. Please also use arrows to indicate cells belonging to each counting category.

One possibility, as mentioned above, is that the homeostasis and post-amputation experiments were performed separately and thus *ets-1*(RNAi) occurred at different penetrance in the two experiments.

However, we do see a fair amount of signal for *calamari* by FISH, which is more evident when the channels are separated. We added arrows to indicate cells of each category (see new Figure 4F).

13. With the current available single-cell dataset, can authors better comment on/suggest the identity of the different glial populations shown in fig.5B-F?

There is a fair amount of data now that there are multiple states of glia after injury, after gene perturbation, or even within uninjured animals (Roberts-Galbraith, et al, *eLife* 2016; Wang, et al, *eLife* 2016; and this manuscript). The published single-cell sequencing datasets do not explore distinct cell types or states within the glial population. We are interested in exploring the nature of these changes in state or identity in our future work.

14. Are 12% and 28% gene expression changes shown in fig.S6B sufficient to lead to pigmentation changes in planarians?

We do see very subtle changes in pigmentation in planarians after *ets-1(RNAi)*, as was seen in He, et al *Development*, 2016. He and colleagues did show some reduction of pooled markers (including *pgbd-1*) by FISH, but did not look at these markers separately or via RT-qPCR. Ets-1 was also shown to have a greater effect on punctate pigment cells than dendritic pigment cells in the analysis in He, et al. Perduring pigment cells are likely to be responsible for the maintained brown pigment after *ets-1(RNAi)*.

15. Please provide FISH images of gad in fig. 6.

We provided the FISH images for *gad* here but we found that it was clearer to show the disorganized *gad* patterning in *ets-1*(RNAi) samples using colorimetric ISH.

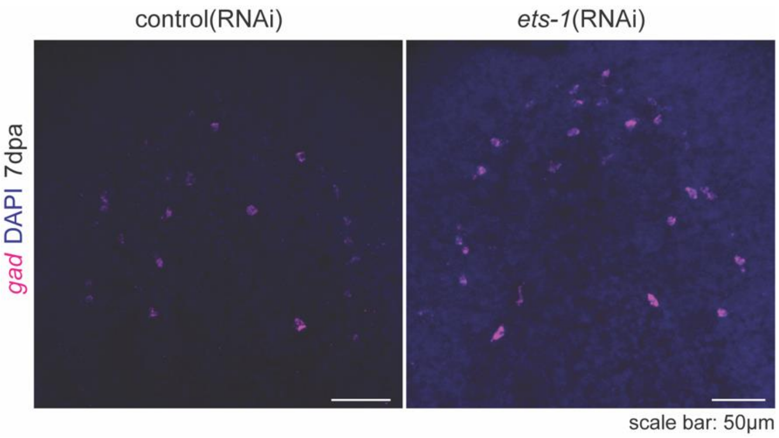

16. Please use the arrow to indicate cells belonging to each counting category for fig. 6H, fig. S7A & B.

We added arrows to indicate cells of each category (see new Figure 4F).

17. The authors mentioned on page 16, that “We did not see phenotypes that would indicate widespread neuronal cell death or failure to specify neurons during regeneration after reduction in glial number.”. Please provide data to support this statement.

© 2023. Published by The Company of Biologists under the terms of the Creative Commons Attribution License (https://creativecommons.org/licenses/by/4.0/).

We do not see reduction of neural cell numbers in the brain, or corresponding behavioral phenotypes like paralysis. We clarified what we meant by this statement in the text.

18. In figure 7, the authors showed interesting locomotion defects of planarians fed with ets-1 RNAi. Beyond the impairment of neuronal function suggested by the authors in the manuscript, it is also very likely due to muscle defects in animals. Can the author exclude this possibility by evaluating the effect of ets-1 RNAi on muscles?

We do not see paralysis or slow body movements as would be expected with muscle defects. We currently do not have a way to evaluate muscle function in planarians, and if we saw a difference in muscle function, it would be impossible to determine whether the difference was due to muscle or neuronal defects.

Reviewer 2 Comments for the Authors:
In this well-written paper, Chandra and colleagues address the development, regeneration, and function of glia in the planarian S. mediterranea. In humans and more widely studied models, glia play important roles in nervous system development and function, but also have inhibitory roles in regeneration by promoting formation of a “glial scar.” Understanding glial biology in systems like planarians that do not form scars is therefore potentially important for regenerative medicine. Prior to this work, multiple markers for planarian glia had been identified, and superficial efforts had identified ets-1 as a potential regulator of glia differentiation, but the functional consequences of glia loss had not been investigated.

First, Chandra et al. demonstrated that glia differentiation occurred after neuron development, by analyzing published RNA-Seq data, and by conducting an elegant and thorough series of in situ hybridization experiments on both regenerating planarians and planarian embryos and hatchlings. They then showed that inhibiting differentiation of eye neurons during homeostasis and regeneration also delayed glia development, and that inhibiting midline and marginal sensory neuron differentiation caused a modest (but statistically insignificant) decrease in glia. Next, they convincingly showed (using multiple markers) that knocking down ets-1 inhibited glia differentiation, and that this affected estrella+ and calamari+ glia subpopulations differently. They went on to show that ets-1 RNAi disrupted neural gene expression and organization, although no specific neuron type seemed to be reduced in number, and many trends were not statistically significant. Finally, ets-1 RNAi also caused behavioral defects, including increased inchworming movement and decreased negative phototaxis.

The authors conclude that the sequence of neurogenesis followed by gliogenesis is conserved in planarians, that (sensory) neurons might be required for gliogenesis, that ets-1 plays a conserved role in gliogenesis, and that glia subtly influence neural gene expression, CNS organization, and neural function. With the caveat that some of the effects of ets-1 inhibition might be due to dysregulation of other phagocytic cell types (poorly understood cathepsin+ cells), the study is the first to identify functional role(s) for planarian glia, building on the PI’s and others’ previous work. This sets the stage for multiple future directions, which the authors outline in their discussion. The work is a novel and substantial advancement in understanding of glia in an emerging regeneration model. However, the authors’ conclusions are somewhat tentative, based on data with small sample sizes and/or statistically insignificant trends. Also, the relationship between the behavior phenotypes observed and the effects on neuronal subpopulations is unclear. The conclusions could be strengthened by addressing these issues in several ways.

We are very grateful for the enthusiasm and support of the reviewer and we appreciate the constructive suggestions for our work. We provide details on the additions and modifications we have made to the manuscript based on the reviewer’s suggestions below.

Major recommendations:
1. Provide more insight into whether inhibiting glia birth/function affects neuron numbers.
In Figure 6A, ets-1 inhibition reduces brain size by about 20%. However, expression of gad, th, and tph (neuron subtype markers) increased by qPCR, gad+ cell numbers did not change but their locations were abnormally patterned, and th+ cell numbers increased significantly in PNS, and insignificantly in CNS/pharynx. This leads to the tentative conclusion (p. 16) that “inhibition of ets-

© 2023. Published by The Company of Biologists under the terms of the Creative Commons Attribution License (https://creativecommons.org/licenses/by/4.0/).

1, *possibly* due to loss of glial cells, *broadly but subtly* impacts planarian neuronal gene expression and CNS organization." While this is true, the data do not explain why the brain might be smaller, or why ets-1 inhibition yields behavioral phenotypes.

We appreciate this critique and have included additional data, quantification, and statistical analysis as detailed below to strengthen and solidify our claims regarding the impact of glia on neurons.

The authors should consider:
- Assessing the reciprocal effect of ets-1 knockdown on photoreceptor cell numbers (ovo/sans/foxQ2/myoVIIA+ cell numbers). Are smaller eyes/fewer photoreceptors responsible for reduced light responsiveness?

We assessed area of the eyespots and found no significant difference in eye spot size after *ets-1(RNAi)*. See new Supplemental Figure 9B.

- Assessing the reciprocal effects of ets-1 knockdown on sensory neuron cell number (soxB1-2+, pkd2L-1+, pkd1L-2+ cell numbers). Are fewer sensory neurons responsible for reduced light responsiveness or motility?

We assessed the number of *pkd1L-2/2L-1* sensory neurons after *ets-1(RNAi)* and determined that there was no significant difference in *pkd*$^{+}$ sensory neuron numbers (with the exception of a slight change in medial *pkd*$^{+}$ cells in the homeostatic trunk). See new Supplemental Figure 9D-H.

- Assessing serotonergic neuron numbers (pitx+, lhx1/5-1+, etc.) since genetic ablation of these neurons also can cause inchworming phenotypes.

We assessed the number of serotonergic neurons with *tph in situ* hybridization and determined that *tph*$^{+}$ cell numbers are not affected after *ets-1(RNAi)*. (See new Figure 5G-J.)

- Assessing markers for other neuronal subtypes.
While doing all of these experiments is probably beyond the scope of the manuscript, determining whether ets-1 reduces CNS/PNS neuron numbers, especially those that might be linked to the observed behavior phenotypes, would strengthen the manuscript considerably.

We took the reviewer's critique to heart and assessed the number of *spp12*$^{+}$, *npp3*$^{+}$, and *tph*$^{+}$ neurons with ISH and determined that there are not significant differences in neural numbers after *ets-1(RNAi)*. (See new figures: Figure 5G-J, P-T; Supplemental Figure 7E-F).

We also revisited our work on dopaminergic neurons and found, consistent with our ISH results, that fluorescent intensity after *tyrosine hydroxylase* FISH was dramatically increased in both the CNS and PNS after *ets-1(RNAi)* (see new Figure 5L). We then repeated quantification of dopaminergic neuron numbers in the PNS, adjusting the intensity of control animal FISH to make the images more comparable. We found there was no difference in *th*$^{+}$ cells in the CNS, but there were still more *th*$^{+}$ cells in the PNS. Thus, the difference in *th* levels by RT-qPCR likely reflects different neural numbers *and* levels of mRNA within single neurons. We corrected this in the main text (See new Figure 5K-O).

While not provided in the manuscript, we show here that there is a nonsignificant increase in fluorescent intensity in gad expression in *ets-1*(RNAi) animals compared to control:

© 2023. Published by The Company of Biologists under the terms of the Creative Commons Attribution License (https://creativecommons.org/licenses/by/4.0/).

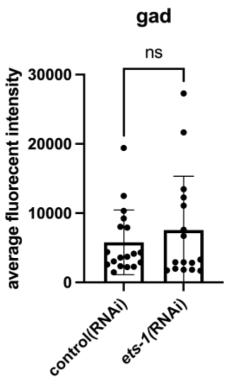

n.s. = not significant (unpaired T-test with Welch's correction)

Taken together, we can solidify claims that *ets-1(RNAi)* and glial depletion does *not* correspond to a change in neuronal numbers in the CNS in our experiments. We therefore interpret changes in mRNA levels to be a change in gene expression within specific neurons (gad, tph), potentially as a result of altered signaling in the microenvironment of the neuron.

2. Provide more insight into whether inhibiting neurogenesis affects gliogenesis. On page 9, section 2 of Results, the authors suggest that "Glial regeneration in sensory structures *might* depend on neurons." It seems that this tentative conclusion is based on the fact that although there was a significant reduction in eye glia, there was only a "consistent but nonsignificant decrease in regeneration of PNS glia." While it seems possible there is a decrease during homeostasis, a downward trend is questionable in the regeneration data (Fig. 3C). This could be addressed in one or more of the following ways, although there may be other methods:

- Quantify more animals/regions or larger areas to reduce variability.

We repeated this experiment with higher Ns and found a significant decrease in *estrella*$^+$ cells that were in contact with *pkd*$^+$ sensory neurons in the homeostatic tissue but not the regenerated tissue. We also assessed more regulators of neurons to see if glial cells were affected in other areas, and revised our previous statement (See Figure 3; supplemental Figure 2).

- Assess glia numbers later, when estrella+ cells should be more abundant (Fig. 1).

Given our current resources, we found it challenging to quantify *estrella*$^+$ cells at later time points due to the difficulty of separating individual estrella$^+$ cell processes intermingle in the CNS and, to a lesser extent, the PNS.

- Use calamari or another marker to quantify glia, since it seems that calamari+ cells are about twice as abundant (Fig. 5B-C).

We do not see expression of *calamari* on the dorsal side of the animal or near the eye. We expect that glia in these locations are *cali*$^-$ or express much less of the mRNA, making this experiment very difficult to perform or interpret.

- Analyze brain regions in soxB1-2 animals, since soxB1-2 reduces brain size (Ross et al., 2018), and the authors were able to quantify brain glia in Fig. 5. These samples might already be available.

We evaluated glial numbers in the CNS in *soxB1-2(RNAi)* animals and found a significant decrease in *estrella*$^+$ cells. (See new Supplemental Figure 2D-F)

- Extend analysis to CNS (brain/VNC) neurons (RNAi of nkx2.1, tcf-1, etc.).

We evaluated glial numbers after *coe(RNAi)* and *sim(RNAi)* and found a significant decrease in *estrella*$^+$ cell numbers. (See new figures 3A-C).

© 2023. Published by The Company of Biologists under the terms of the Creative Commons Attribution License (https://creativecommons.org/licenses/by/4.0/).

- Knock down nou darake or tec-1 to increase neuron numbers. Would this be accompanied by more glia?

We evaluated glial numbers after *nou-darake(RNAi)* and found that in the expanded posterior region of the brain, there was an increase in *estrella*$^{+}$ cells (See new figures 3D-F).

Minor recommendations:
1. ets-1 knockdown seemed to have modest but insignificant effects on photoreceptor axons/connectivity (Fig. S7G-J). If the total number of defects per animal is added together, does a phenotype become more obvious/significant?

We repeated our analysis in the way suggested and found that, indeed, *ets-1(RNAi)* animals exhibit significantly more cases of abnormal axon connectivity compared to control. See new Figure 6A-B; Supplemental Figure 8A-D.

2. The authors stated (p. 16) that “we did not see phenotypes that would indicate widespread neuronal cell death…” without specifying what phenotypes would indicate this, since they only assessed a limited number of neuronal markers and did not assess cell death directly. Could the authors clarify what phenotypes would indicate widespread death?

We do not see reduction of neural cell numbers in the brain, or corresponding behavioral phenotypes like paralysis. We clarified what we meant by this statement in the text.

3. The abstract states that “…ets-1(RNAi) impairs nervous system architecture…” but the title of Figure S7 is “ets-1 knockdown does not affect neural architecture.” These statements conflict.

We clarified and reconciled these statements in the text. We conclude that *ets-1(RNAi)* does affect nervous system architecture but not *neuronal* cell numbers.

4. It is not clear how many days elapsed after feeding and amputation before the authors assessed the dependence of glial cell regeneration on neurons (Figure 3). The authors could consider adding an RNAi timeline as in Figure 4C to Figure 3 or clarify in the figure legend.

We added this detail within the methods section and within the figures.

5. Obvious examples of inchworming are not apparent in the videos. Can the authors provide a higher magnification example?

We included stills from the videos (see new Figure S9C) and also described the phenotype in more detail. We see inch worming and also worms starting/stopping and casting their heads side to side. The phenotypes together might represent decreased coordination of and commitment to movement.

Reviewer 3 Comments for the Authors:
This study by Chandra et al explores the role of planarian glial during regeneration and the function of the transcription factor Ets-1 during glial cell regeneration in the planarian Schmidtea mediterranea. The authors show glial cell development and regeneration occur after the generation and regeneration of neurons and suggest that neurons are required for the emergence of glial cells. They show that Ets-1 is required for the maintenance of existing glial cells in uninjured tissues and for the regeneration of new glia after injury. They conclude that ets-1 RNAi non-cell-autonomously impacts neuronal gene expression, neuropil size, and animal movement.

This is a well-executed and significant study that explores the role of glial cells and the functional relationship between glia and neurons during development and regeneration. This reviewer has only few suggestions focused on data analysis and presentation that will improve the strengths of the study.

We are very grateful for the reviewer’s constructive feedback and excitement about our work. We have incorporated the suggestions of the reviewer as detailed below.

© 2023. Published by The Company of Biologists under the terms of the Creative Commons Attribution License (https://creativecommons.org/licenses/by/4.0/).

1-The authors used ovoRNAi to disrupt neurogenesis and to show that neurons are required for glial development and regeneration. The evidence for this conclusion is insufficient and inconclusive.

Quantifications in figures 3B and S2A are less convincing in the absence of error bars and N numbers. Normalized quantifications and data presentation similar to the quantifications in 3C are needed to support the conclusion.

We did not provide error bars in this figure since the data are categorical (presence or absence of cells) and compiled. However, to address the larger concern of the reviewer, we have repeated this experiment with higher N and separated the individual categories of eye loss for easier understanding. New data are included in Figures 3H, Supplemental Figure 2A-B.

In addition, it is unclear why nearly 1/3 of the scored eyes had no estrella expression in the control RNAi condition although 100% of the control eyes were positive for sans. How much of these quantifications depend on a user defined threshold for Estrella or sans expression level? Quantifying normalized fluorescence signal in addition to binary scoring of cells as positive or negative would address this concern. If confirmed, how do the authors interpret the lack of estrella expression in a subset of the control RNAi sample.

We repeated the experiment with higher N and separated individual categories for a more thorough analysis. Please see new data in Supplemental Figures 2A-B.

2-The effects of ets-1 on glial cells is remarkable, but opens a question about the differential roles of calamari and estrella in different glial cell populations. The data suggest that calamari and/or calamari expressing cells are the main targets of ets-1. What is known about calamari versus estrella and the cells they express? In light of the data presented in figure 5, it would be informative to check the expression of calamari after ovoRNAi. Could calamari expressing cells be more sensitive to the generation and regeneration of neurons?

We do not see expression of *calamari* on the dorsal side of the animal or near the eye. We expect that glia in these locations are *cali*$^{-}$ or express much less of the mRNA, making this experiment very difficult to perform or interpret.

3-Figures 1 and 2 include nice ISH showing that development and regeneration of neurons precede glia. Including error bars and statistics in these quantifications would help strengthen this conclusion.

We provided number of animals that exhibited these phenotypes (see new Figures 1A and Supplemental Figure 1).

4-The effects of glial cells on neuron gene expression is intriguing and I agree with the authors that additional future studies are needed to better understand the gene expression changes observed in neurons. However, and although ets-1 has been previously studied in glial cells, the study did not establish that manipulating ets-1 results in “non-autonomous” effects on neurons. Detailed expression analysis for
ets-1 in neurons and glia beyond what is shown in figure 4A will help address this question.

ets-1 is strongly expressed in *cathepsin*$^{+}$ progenitors and cell types in the planarian, but not in neurons or neural progenitors based on single-cell sequencing datasets (Fincher, et al *Science* 2018; Plass, et al *Science* 2018). Ets-1 was also shown to be co-expressed with *foxF-1* and present in differentiating stem cells in Dubey, et al 2022 *Journal of Cell Science*.

5-The effects of glial cells on animal behavior is also of interest. The methods section states that experiments were performed for 3 biological replicates. But it is difficult to appreciate how variable of consistent behaviors are in 7A in the absence of error bars or statistics. It is also important to normalize the data in 7B to show % of animals per replicate with error bars instead of or in addition to number of animals.

© 2023. Published by The Company of Biologists under the terms of the Creative Commons Attribution License (https://creativecommons.org/licenses/by/4.0/).

We had previously provided information on all replicates (originally Supp. Fig. 8; see new Supp. Fig 9A-C). However, to address the reviewer's concern, we added quantification from 660 second timepoint with statistics and show that there is a significant difference in *ets-1*(RNAi) animals remaining in the light side compared to *control(RNAi)* animals.

6-The study clearly shows a role for ets-1 during glial cell maintenance in figure 4 but those findings are not quantified.

We repeated experiments in uninjured animals and show *ets-1*(RNAi) affects glial cell maintenance. Compared to control, we observed that there is a robust loss of *calamari* expression in intact animals, but more subtle decrease in *estrella* expression (in contrast to regenerated animals) (See new Figures 4F-L).

7-The RNAi Methods section states "The number of animals alive was quantified as detailed in Fig S3A". I believe this refers to figure S5.

We made this correction in the text.

Second decision letter

MS ID#: DEVELOP/2023/201666

MS TITLE: Ets-1 transcription factor regulates glial cell regeneration and function in planarians

AUTHORS: Bidushi Chandra, Matthew G Voas, Erin L Davies, and Rachel Roberts-Galbraith
ARTICLE TYPE: Research Article

I am happy to tell you that your manuscript has been accepted for publication in Development, pending our standard ethics checks.

Reviewer 1

Advance summary and potential significance to field

The author's response resolved some of my concerns about this paper. The clarification on the specificity of RNAi reduced my concerns about this method (The authors did discuss the impact of ets1 RNAi on pigment and other cathepsin+ cells and acknowledge the potential role of these cells on the maintenance and regeneration of CNS system). Including intact animals in the RNAi experiments clarifies the role of ets1 in maintaining glial cells and synapse organization during homeostatic conditions. It is overall a successful attempt to study the interrelationship between gliogenesis and neurogenesis in planarians.

Comments for the author

The authors have addressed most of my comments by clarifying their writing or performing additional experiments. The authors clearly stated things that they are pursuing, which can be out of the scope of the revision for this manuscript. Overall, the paper has been improved and can be considered for publication in Development.

© 2023. Published by The Company of Biologists under the terms of the Creative Commons Attribution License (https://creativecommons.org/licenses/by/4.0/).

Reviewer 2

Advance summary and potential significance to field

The addition of new RNAi data (coe, sim, and ndk) have added considerable new support for the idea that neurogenesis precedes and is required for gliogenesis. The rigorous assessment of multiple additional neuronal subtypes suggests that smaller brain size is indeed due to fewer glia and/or neuropil disorganization, and not fewer neurons. Finally, the increased sample sizes have strengthened quantitative analysis throughout the paper.

Concerns have been thoroughly addressed. This is a well written and rigorous contribution to our understanding of glial biology and nervous system regeneration.

Comments for the author

At the authors' discretion, it might be helpful to add one or more arrows to the locomotion videos pointing out inchworming behavior, especially for readers not familiar with planarians.

Reviewer 3

Advance summary and potential significance to field

This is a well-executed study that explores the role of glial cells and the functional relationship between glia and neurons during development and regeneration. The study opens up questions and future avenues to better understand the role of glial cells in planarians.

Comments for the author

The authors have addressed all my comments.

© 2023. Published by The Company of Biologists under the terms of the Creative Commons Attribution License (https://creativecommons.org/licenses/by/4.0/).
